# Genome-wide transcriptomic analysis uncovers the molecular basis underlying early flowering and apetalous characteristic in *Brassica napus* L

**DOI:** 10.1038/srep30576

**Published:** 2016-07-27

**Authors:** Kunjiang Yu, Xiaodong Wang, Feng Chen, Song Chen, Qi Peng, Hongge Li, Wei Zhang, Maolong Hu, Pu Chu, Jiefu Zhang, Rongzhan Guan

**Affiliations:** 1 State Key Laboratory of Crop Genetics and Germplasm Enhancement, Nanjing Agricultural University, Nanjing, 210095, China; 2 Key Laboratory of Cotton and Rapeseed, Ministry of Agriculture/Institute of Industrial Crops, Jiangsu Academy of Agricultural Sciences, Nanjing, 210014, China

## Abstract

Floral transition and petal onset, as two main aspects of flower development, are crucial to rapeseed evolutionary success and yield formation. Currently, very little is known regarding the genetic architecture that regulates flowering time and petal morphogenesis in *Brassica napus*. In the present study, a genome-wide transcriptomic analysis was performed with an absolutely apetalous and early flowering line, APL01, and a normally petalled line, PL01, using high-throughput RNA sequencing. In total, 13,205 differential expressed genes were detected, of which 6111 genes were significantly down-regulated, while 7094 genes were significantly up-regulated in the young inflorescences of APL01 compared with PL01. The expression levels of a vast number of genes involved in protein biosynthesis were altered in response to the early flowering and apetalous character. Based on the putative rapeseed flowering genes, an early flowering network, mainly comprised of vernalization and photoperiod pathways, was built. Additionally, 36 putative upstream genes possibly governing the apetalous character of line APL01 were identified, and six genes potentially regulating petal origination were obtained by combining with three petal-related quantitative trait loci. These findings will facilitate understanding of the molecular mechanisms underlying floral transition and petal initiation in *B. napus*.

The emergence of flowers as reproductive units probably contributed substantially to the evolutionary success of flowering plants. In the life cycle of an angiosperm plant, the transition from vegetative to reproductive development is tightly controlled by a complex gene regulatory network. Over the past three decades, work in *Arabidopsis thaliana*, as well as in several other angiosperm species, including snapdragon (*Antirrhinum majus*), petunia (*Petunia hybrida*) and rice (*Oryza sativa*), has identified a vast number of genes involved in floral transition^1^,^2^,^3^. Recently several reviews provided detailed insights into the gene regulatory network underlying floral transition, which mainly consists of vernalization, photoperiod, gibberellins (GAs), autonomous, ambient temperature and aging pathway^1^,^2^,^3^. The genetic circuits that integrate different signals eventually converge to activate the expression of a group of so-called floral meristem (FM)-associated genes, including *LEAFY* (*LFY*) and *APETALA1* (*AP1*)^1^,^2^,^4^,^5^,^6^. The floral organ-associated genes are subsequently activated by *LFY* and *AP1*, FM develops into distinct domains that give rise to the different types of floral organs^7^,^8^.

Floral organ morphogenesis, not in only the model plants *A. thaliana* and *A. majus*, and the model of floral organ specifications become increasingly clear in the basal angiosperm^9^,^10^,^11^,^12^. According to the ‘quartet model’ of petal specification in *Arabidopsis*, seven floral organ-associated genes, *AP1*, *AP3*, *PISTILLATA* (*PI*), *SEPALLATA 1* (*SEP1*), *SEP2*, *SEP3* and *SEP4*, encoding MADS-box transcription factors are specifically expressed in conjunction with each other in the second whorl and specify the petal’s identity^13^,^14^. Evolution studies indicate that B function genes underwent two vital duplication and divergence events that orderly generated the *PI*, paleo*AP3*, eu*AP3* and TM6 lineages, and the appearance of eu*AP3* lineage was closely related to petal origin in higher eudicots^15^,^16^. In addition, there are at least 94 genes involved in petal development in *Arabidopsis*, and a majority of these genes were highly involved in A, B or E-class gene expression. Interestingly, a few of the genes functioning in floral transition appear to play roles in petal development, such as *AINTEGUMENTA-LIKE 5* (*AIL5*) and *TOUSLED* (*TSL*)^17^,^18^.

Rapeseed (*Brassica napus*, AACC, 2n = 38) is an allotetraploid crop that was formed ~7500 years ago by the hybridization between *Brassica rapa* (AA, 2n = 20) and *Brassica oleracea* (CC, 2n = 18) as well as by chromosome doubling^19^. A comparative evolutionary analysis revealed that *B. napus* had a common ancestor and a high degree of chromosomal colinearity with *Arabidopsis* because the progenitors diverged about 20 million years ago^20^,^21^. Anthesis, as a key adaptive trait, is crucial to rapeseed yield. Early flowering ensures oil production to some extent, in winter oilseed rape by avoiding high temperature stress during the mature period. Although a myriad of quantitative trait loci (QTLs) associated with flowering time were detected in prevision studies, only a few flowering genes were identified in *B. napus* through sequence homology analysis, including *FLOWERING LOCUS T* (*FT*), *CONSTANS* (*CO*), *FLOWERING LOCUS C* (*FLC*), *SUPPRESSOR OF OVEREXPRESSION OF CONSTANS 1* (*SOC1*) and *FRIGIDA* (*FRI*)^22^,^23^,^24^,^25^,^26^. The molecular basis that underlies the regulation of flowering time is poorly understood in *B. napus*.

Apetalous rapeseed with floral organs that are fully developed, except petals, is considered the ideotype of high-yield rapeseed because of its low-energy consumption, high photosynthetic efficiency and superior klendusity to *Sclerotinia sclerotiorum*^27^,^28^,^29^,^30^,^31^,^32^. Unlike all of the apetalous mutants in *Arabidopsis* and *Antirrhinum*, the number and morphology of sepals, stamens and carpels of many apetalous rapeseeds detected in earlier studies are similar to those of the natural variety^33^,^34^, seemingly indicating that the genetic mechanism governing petal development of rapeseed is not completely consistent with the model plants at some level. However, the genetic analysis of the apetalous characteristic of *B. napus* is insufficient because very few stable apetalous mutants are generated. A few studies suggested that the apetalous characteristic in *B. napus* is governed by recessive genes, possibly by two to four loci^35^, and identified several associated with QTLs^33^,^34^. Only one study suggested that there are two types of *AP3* genes in *B. napus*, *B. AP3.a* and *B. AP3.b*^36^. A knockdown of *B. AP3.a* led to a deficiency of petals, while natural expression of *B. AP3.b* ensured normal stamen morphogenesis^36^. However, the theory failed to explain the determination of the correct number of sepals. Thus, the mechanism underlying the apetalous characteristic of rapeseed appears to be more complex than initially believed. Fortunately, the genome sequence of *B. napus* was released in 2014^19^ and will contribute to the detection of floral regulatory genes in the whole genome using bioinformatics.

RNA sequencing (RNA-seq) as a revolutionary tool for transcriptomics has been broadly used to explore the molecular basis governing the phenotypic traits of organisms^37^. In the present study, the rapeseed lines APL01 and PL01, two lines with distinguishable flowering time and petal morphologies, were used for Illumina RNA-seq to study the differential expressed genes (DEGs) in the young inflorescences. In combination with gene ontology (GO)-enrichment analysis and homologous alignments, the discovery of the molecular basis underlying early flowering and apetalous characteristic in line APL01 is expected. Meanwhile, the detection of potential candidate genes regulating the petalous degree (PDgr) of rapeseed is expected to be assisted by coupling RNA-seq with QTL mapping.

## Results

### Phenotypic characteristics comparison between lines APL01 and PL01

**Figure 1:**
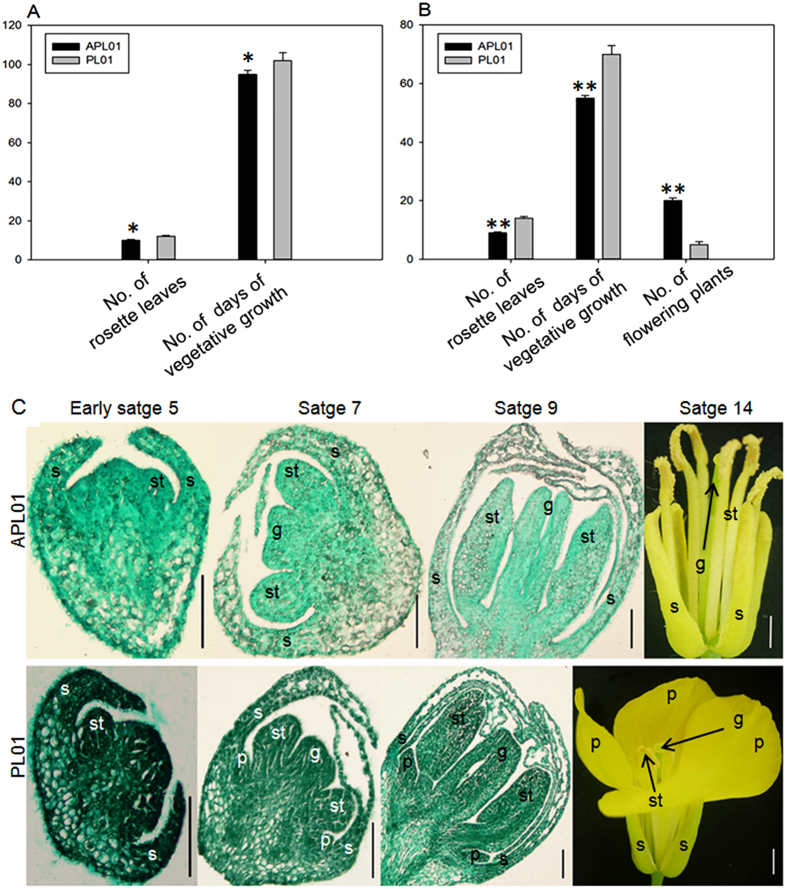
Characterization of flowering time and petal morphogenesis in lines APL01 and PL01. (**A**) The number of rosette leaves and days of vegetative growth in APL01 and PL01 at the beginning flower stage in the field. (**B**) The number of rosette leaves, days of vegetative growth and flowering plants in APL01 and PL01 at the beginning flower stage in the greenhouse. (**C**) Buds at early stages 5, 7 and 9, and flowers at stage 14 in APL01 and PL01. Single asterisk indicates that the difference is significant (non-paired *t*-test, *P* < 0.05), double asterisks indicate that the difference is extremely significant (non-paired *t*-test, *P* < 0.01). s, sepal; p, petal; st, stamen; g, gynoecium. Black bar = 100 μm, white bar = 2 mm.

Flowering time is the first differentiating characteristic between lines APL01 and PL01 during the blossoming period. The anthesis of line APL01 is five days earlier (non-paired *t*-test, *P* < 0.05) than line PL01 in the field (Fig. 1A, Supplementary Fig. S1A). In the greenhouse, all 20 plants of line APL01 were beginning to blossom on the 55^th^ day after sowing, however, only five plants of line PL01 had flowered by the 70^th^ day after sowing, and the remaining plants failed to bloom, perhaps due to a vernalization failure (Fig. 1B, Supplementary Fig. S1B).

Another remarkable characteristic of line APL01 is its complete apetalous status, while line PL01 is normally petalled (Fig. 1C, Supplementary Fig. S1C). To dissect the aberrance of line APL01, the early flower development of lines APL01 and PL01 were observed using paraffin sections. As shown in Fig. 1C, the early flower development of lines APL01 and PL01 is the same, except for petal morphogenesis. The appearance of petal primordia in line PL01 occurs later than stamen primordia, while petal primordia of line APL01 don’t arise in the second whorl all of the time (Fig. 1C), implying that the apetalous characteristic of line APL01 is determined at the initial petal primordia stage (later in stage 5).

### Analysis of gene expression in the young inflorescences from lines APL01 and PL01

Because the variations in flowering time and petal morphogenesis are dominantly based on gene expression changes that occur before the initiation of FMs and petal primordia, young inflorescences only comprised of shoot apical meristem (SAM) and buds at stages 1 to 5 (Based on the summary of stages of flower development in *Arabidopsis*^38^), were collected from lines APL01 and PL01 for high-throughput RNA-seq. In total, 56.01 to 69.38 million raw reads for each sample were generated, and three biological replicates for each line were performed (Table 1). Subsequently, 55.31 to 68.42 million clean reads were generated by removing low quality regions and adapter-related sequences, and were mapped to the *B. napus* genome using TopHat2^39^ (Table 1).

**Figure 2:**
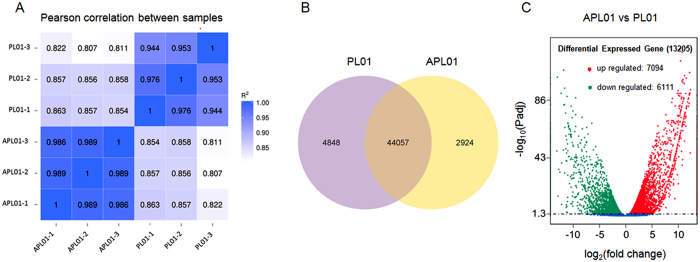
Comparison of gene expression levels in the young inflorescences of lines APL01 and PL01. (**A**) Pearson correlation coefficients among gene expression levels in six samples, with R^2^ > 0.8 as the significance threshold. (**B**) Venn diagram of genes expressed (RPKM > 1) in the young inflorescences of lines APL01 and PL01. (**C**) Volcano plot of DEGs in the young inflorescences of line APL01 compared with those of line PL01, with −log(padj) > 1.3 as the significance threshold.

Additionally, we evaluated the gene expression levels that were expressed [reads per kilo base per million (RPKM) > 1] in lines APL01 and PL01 with HTSeq^40^, and then analyzed the Pearson correlation between six samples (Fig. 2A, Supplementary Fig. S2). As shown in Fig. 2A, Pearson correlation coefficients (R^2^) between three biological replicates for each line are greater than 0.94 all of the time, indicating that samples from each line are available. The genetic variation between lines APL01 and PL01 is seemingly small (R^2^ > 0.8) (Fig. 2A). In total, 44,057 genes were expressed (RPKM > 1) in both lines APL01 and PL01, 2,924 genes were specifically expressed in line APL01 and 4848 genes were specifically expressed in line PL01 (Fig. 2B).

Further more,13,205 DEGs (adjusted *P* value < 0.05) were identified by the DESeq R package^41^, in which 7094 genes were significantly up-regulated and 6111 genes were significantly down-regulated in the young inflorescences of line APL01 as compared with those of line PL01 (Fig. 2C, Supplementary Data 1).

### Assessing RNA-seq results by quantitative real time RT-PCR assay

**Figure 3:**
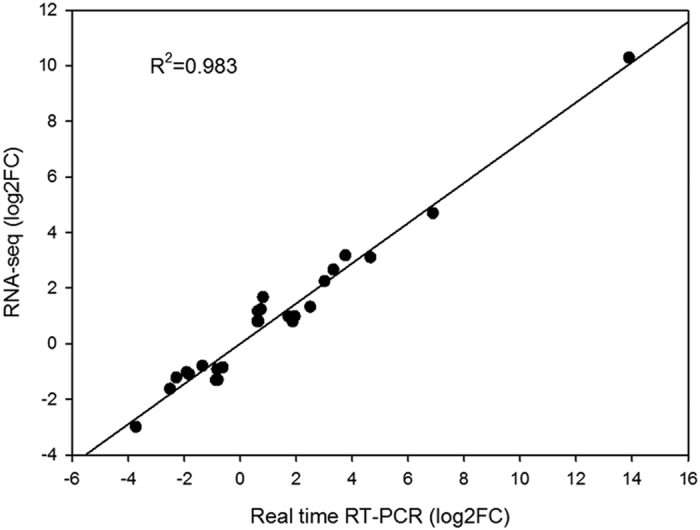
Validation of the expression data from RNA-seq assay by qRT-PCR. Twenty seven DEGs from the RNA-seq assay were used for qRT-PCR assay. Pearson’s correlation between RNA-seq data and qRT-PCR data is unexceptionable, with R^2^ > 0.8 as the significance threshold.

To evaluate the reliability of RNA-seq results, the expression patterns of 27 DEGs identified in the RNA-seq assays were verified by quantitative reverse transcription-PCR (qRT-PCR). The subsequent results suggested that 10 genes showed at least a 1.5-fold (log2FC = −0.62) down-regulation, while 17 genes displayed a more than 1.5-fold (log2FC = 0.61) up-regulation in the inflorescences of line APL01 compared with those of line PL01 (Fig. 3, Supplementary Table S1). Furthermore, all 27 genes showed the same expression pattern in the qRT-PCR assays as in the RNA-seq data (Fig. 3), suggesting that RNA-seq data is reliable. In addition, the Pearson correlation coefficient between qRT-PCR data and RNA-Seq data was 0.983 (p = 2.70E-04), further confirming the validity of the RNA-seq data (Fig. 3).

### DEGs involved in protein biosynthesis accompany early flowering and the apetalous characteristic

To understand gene functions related to early flowering and the apetalous character of line APL01, a GO enrichment analysis for the DEGs was performed using the GOseq R package^42^. The relationships among the significantly enriched GO terms are shown through a directed acyclic graph (DAG) (Supplementary Fig. S3).

**Figure 4:**
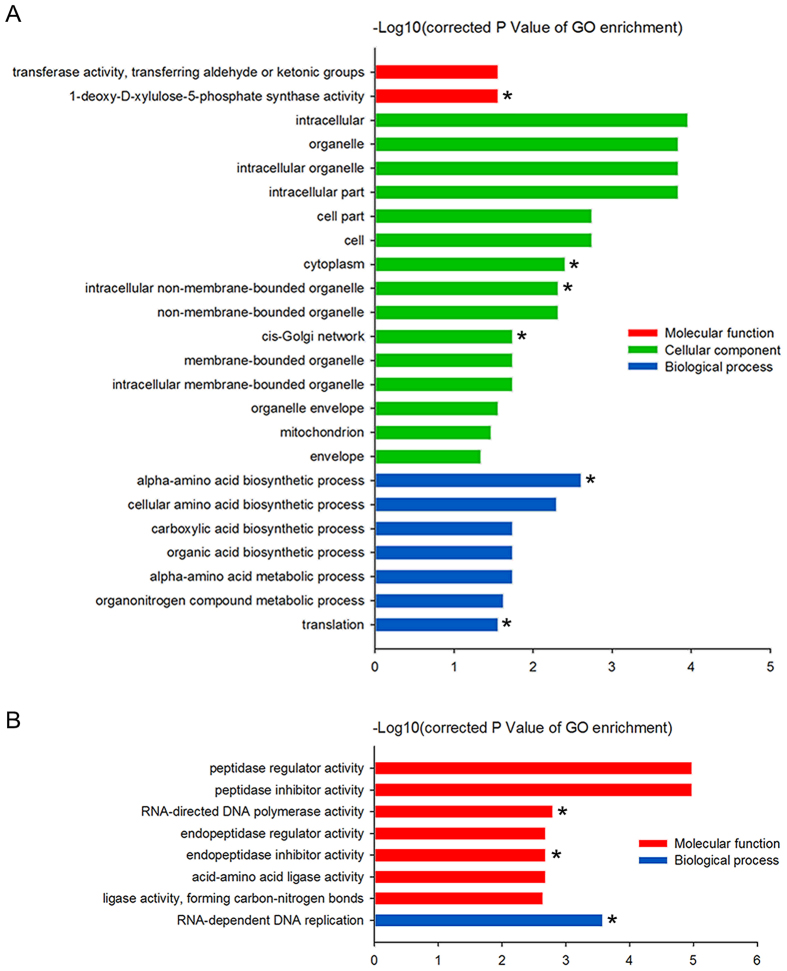
GO terms (corrected *P* value < 0.05) significantly enriched by DEGs in the young inflorescences of line APL01 vs line PL01. (**A**) Significantly enriched GO terms in the down-regulated genes. (**B**) Significantly enriched GO terms in the up-regulated genes. ‘Molecular function’, ‘cellular component’ and ‘biological process’ categories are shown in red, green and blue, respectively. GO terms indicated with asterisks are the terminal nodes of each DAG. -Log10(corrected *P* value) > 1.30 as the significance threshold. GO terms were sorted based on corrected *P* values.

For the 6,111 down-regulated genes in line APL01, 24 significantly enriched GO terms were identified. Among these GO terms, “alpha-amino acid biosynthetic process” (q = 2.46E-03), “intracellular” (q = 1.11E-04) and “1-deoxy-D-xylulose-5-phosphate synthase activity” (q = 2.76E-02) are the most significantly enriched in the ‘biological process’, the ‘cellular component’ and the ‘molecular function’ groups, respectively (Fig. 4A). In combination with the DAG of the ‘biological process’ group (Supplementary Fig. S3A), we found that abundant numbers of down-regulated genes categorized in the ‘biological process’ group were aggregated in the categories of “alpha-amino acid biosynthetic process” (q = 2.46E-03) and “translation” (q = 2.76E-02). Likewise, the DAG of the ‘cellular component’ group showed that many down-regulated genes categorized in the ‘cellular component’ group were aggregated in the categories of “cytoplasm” (q = 3.94E-03), “intracellular non-membrane-bounded organelle” (q = 4.82E-03) and “cis-Golgi network” (q = 1.81E-02) (Supplementary Fig. S3B). These GO terms are implicated in protein biosynthesis. DEGs involved in these GO terms displayed a more than 1.6-fold (log2FC = −0.72) reduction in line APL01 as compared with line PL01 (Supplementary Data 2).

For the 7094 up-regulated genes in line APL01, eight GO terms were significantly enriched, with the most significantly enriched GO terms being “RNA-dependent DNA replication” (q = 2.66E-04) in the ‘biological process’ group and “peptidase inhibitor activity” (q = 1.06E-05) in the ‘molecular function’ group (Fig. 4B). Based on the DAG of the ‘molecular function’ group (Supplementary Fig. S3C), abundant numbers of up-regulated genes categorized in the ‘molecular function’ group were aggregated in the categories of “acid-amino acid ligase activity” (q = 2.06E-03), “endopeptidase inhibitor activity” (q = 2.06E-03) and “RNA-directed DNA polymerase activity” (q = 1.59E-03). The former GO term promotes protein biosynthesis, while the latter two functional categories competitively impede protein biosynthesis. DEGs related to these GO terms showed 1.6-fold increases in line APL01 compared with line PL01 (Supplementary Data 2).

Notably, protein biosynthesis, as a key component of the basic cellular processes responsible for cell division and differentiation, is necessary for tissue development and expansion. Thus, these findings indicated that the aforementioned DEGs involved in protein biosynthesis were required for the basic cellular processes responsible for floral transition or/and petal onset in *B. napus*, especially those genes only expressed in the young inflorescences of line APL01 or line PL01 (Supplementary Data 2).

### DEGs promote early flowering potentially through vernalization and photoperiod pathways

**Figure 5:**
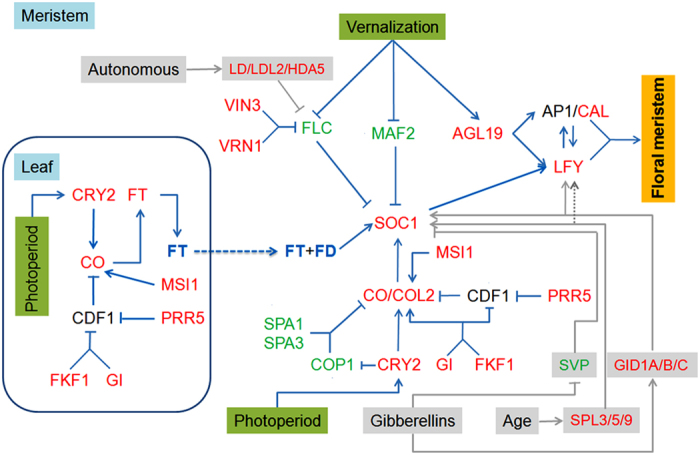
Gene regulatory networks promoting early flowering in line APL01. In the vernalization pathway, *AGL19* acts as an activator of the FM identity genes (*LFY* and *AP1*), while both *MAF2* and *FLC* repress *SOC1* expression; *VIN3* and *VRN1* shut down *FLC* transcription by methylating the histones of the FLC-associated chromatin. For the photoperiod pathway, *CRY2*, as a receptor of blue light, represses *COP1*, which is the E3 ubiquitin ligase ubiquitinating CO. *CO* acts as a key activator of *FT* and *SOC1*, which play vital roles in integrating multiple flowering signals. *SPA1* and *SPA3* bind to *COP1* and regulate CO protein levels. *CDF1* acts as a suppressor of *CO* transcription. *FKF1* represses *CDF1* expression together with *GI*, and promotes *CO* expression. *MSI1* acts upstream of the CO-FT pathway to enable an efficient photoperiodic response. FT protein (blue font), as a florigen transferred from leaf to shoot apex (blue dotted arrow), interacts with the FD protein at the shoot apex and activates downstream targets, such as *SOC1* and *AP1*. In addition, autonomous, GA and aging pathways probably participated in the early flowering of line APL01 (pathways indicated in dark gray). Genes indicated in red are up-regulated, while genes indicated in green are down-regulated in shoot apical meristems or leaves of line APL01 compared with those of line PL01. Genes in black are not significantly changed between two lines, genes indicated in blue represent protein. Arrows represent the promotion or gene activation, and blunted lines represent gene repression. The detailed gene functions are described in Supplementary Table S3.

To discern the regulatory networks underlying the early flowering of line APL01, 1093 putative homologs of *Arabidopsis* 282 flowering genes were identified in the *B. napus* genome through homology alignment (Supplementary Data 3). Based on RNA-seq data, 82 DEGs were possibly involved in the early flowering of line APL01, in which most of the genes functioning in the vernalization and photoperiod pathway were contained (Supplementary Table S2). The possible regulatory network governing the early flowering of line APL01 shown in Fig. 5 is based on the floral transition network in *Arabidopsis*^1^ (Supplementary Table S3).

Among eight vernalization-related DEGs, the putative rapeseed *FLC* (*BnaC09g46540D*), a main suppressor of *FT* and *SOC1*, displayed a 3.8-fold (log2FC = −1.94) decrease in line APL01, which is probably attributed to the up-regulation of *VERNALIZATION INSENSITIVE 3* (*VIN3*) and *VERNALIZATION 1* (*VRN1*). In this study, the putative rapeseed *VIN3* (*BnaA02g08140D* and *BnaA03g10310D*), which shuts down *FLC* transcription by methylating the histones of *FLC’*s chromatin, displayed a more than 2.1-fold up-regulation. Meanwhile, the putative rapeseed *VRN1* (*BnaA03g35020D* and *BnaC01g33680D*), which maintains the methylated state of *FLC’*s chromatin, was also up-regulated at least 5.8-fold in line APL01 (Fig. 5, Supplementary Table S2). In addition, the putative rapeseed *MADS AFFECTING FLOWERING 2* (*MAF2*) (*BnaC03g04170D*), another vital suppressors of *SOC1*, was only expressed in line PL01, while the putative rapeseed *AGAMOUS-LIKE 19* (*AGL19*), a direct activator of the FM identity genes, *LFY* and *AP1*, was elevated 5.9-fold in line APL01, which probably promoted *SOC1* and *LFY* expression (Fig. 5, Supplementary Table S2).

In the photoperiod pathway, the putative rapeseed *SUPPRESSOR OF PHYA-105 1* (*SPA1*) (*BnaA03g21350D*) and *SPA1-RELATED 3* (*SPA3*) (*BnaC01g36140D*), two negative regulators of the CO protein level, were only expressed in line PL01. Together with the down-regulated *CONSTITUTIVELY PHOTOMORPHOGENIC1* (*COP1*) (*BnaA05g34990D*) (log2FC = −0.8), which ubiquitinates CO, the CO protein level and stability in line APL01 was higher than PL01. The putative rapeseed *CO* (*BnaA02g02840D*) and *CONSTANS-LIKE 2* (*COL2*) (*BnaC03g32910D*), two main activators of *FT* and *SOC1*, respectively, were up-regulated 8.6-fold (log2FC = 3.11) and 26-fold (log2FC = 4.7), respectively, in line APL01, which facilitated *SOC1* up-regulation (Fig. 5, Supplementary Table S2). Furthermore, given that *FT*, as the terminal signal integrator of the photoperiod pathway, is dominantly expressed in leaves, the expression patterns of the three *FT* genes (*BnaC02g23820D*, *BnaA02g12130D* and *BnaA07g33120D*) and 15 photoperiod-related DEGs identified by RNA-seq were verified in young leaves from lines APL01 and PL01 through qRT-PCR (Supplementary Fig. S4). In total, the 13 genes display at least 1.5-fold changes in gene expression levels between lines APL01 and PL01, and the putative rapeseed *FT* (*BnaA02g12130D*) had a 344.6 fold higher expression level in line APL01 as compared with line PL01, further confirming that the early flowering of line APL01 is partially due to the photoperiod pathway (Supplementary Fig. S4).

Lastly, the putative rapeseed flowering integrators *SOC1* (*BnaC04g50370D*), floral meristem identity genes *LFY* (*BnaCnng24550D*) and *CAULIFLOWER* (*CAL*) (*BnaC03g56640D*), displayed 1.7-fold (log2FC = 0.79), 1.9-fold (log2FC = 0.93) and 4.2-fold (log2FC = 2.08) elevated levels, respectively, in line APL01 compared with line PL01, implying a higher efficiency in floral transition in line APL01 (Fig. 5, Supplementary Table S2). In addition, a few genes functioning in other flowering pathways also displayed differential expression levels between lines APL01 and PL01 (Supplementary Table S2), implying that these genes possibly regulate early flowering of line APL01, but it needs further research. Whereas, combining with the greenhouse cultivation, our results suggested that the above DEGs functioning in vernalization and photoperiod pathways were potentially involved in the regulation of early flowering of line APL01.

### The upstream genes participate in the regulation of petal morphogenesis in *B. napus*

To explore the molecular basis governing the apetalous characteristic of line APL01, 372 homologs of 94 genes involved in petal development in *Arabidopsis* were detected in the *B. napus* genome (Supplementary Data 4). Combined with RNA-seq results, 36 genes, with expression changes that probably hamper petal development, were identified (Table 2). However, three vital MADS-box transcription factors, AP1, AP3 and PI, regulating petal morphogenesis in angiosperm plants showed no obvious changes in gene expression levels between lines APL01 and PL01.

A further analysis revealed that these 36 genes were involved in transcriptional regulation, epigenetic modification, protein ubiquitination and protein farnesylation (Table 2, Supplementary Table S4). For 16 transcription factors, the genes showed at least 1.8-fold expression changes between lines APL01 and PL01. In particular, the putative rapeseed *AUXIN RESPONSE FACTOR 2* (*ARF2*) (*BnaA05g14370D* and *BnaA06g14090D*), a transcription repressor of cell division and organ growth that mediates gene expression in response to auxin, had a more than 1254.6-fold higher expression level in line APL01 compared with line PL01. Two other positive transcription factors responsible for petal development, the putative rapeseed *PENNYWISE* (*PNY*) (*BnaC03g00520D*) and *SEP2* (*BnaC05g48320D*), a BELL1-like (BELL) homeobox and a MADS-box protein, respectively, which are crucial to normal petal development, were only expressed in line PL01, but no expression in line APL01. Nine epigenetic regulation-related genes displayed more than two fold changes in gene expression levels between the two lines. These genes included the putative rapeseed *SERRATED LEAVES AND EARLY FLOWERING* (*SEF*) (*BnaA10g11890D*), a putative component of a chromatin-remodeling complex negatively regulating petal morphogenesis, that was up-regulated by as much as 126-fold (log2FC = 6.98) in line APL01. Two transcriptional repressors, the putative rapeseed *TOPLESS* (*TPL*) (*BnaA07g19900D*), which restricts petal fate by regulating the outer boundary of B-class gene expression, together with *AP2* and *HDA19*, had a 2.8-fold (log2FC = 1.51) increase, while the putative rapeseed *ASYMMETRIC LEAVES 2* (*AS2*) (*BnaA02g12180D*), which represses boundary-specifying genes for normal petal development, decreased 2.5-fold (log2FC = −1.31) in line APL01. One transcriptional co-activator, the putative rapeseed *MEDIATOR SUBUNIT 8* (*MED8*) (*BnaC09g21160D*), as a subunit of the Mediator complex that positively regulates petal size, was down-regulated 2.4-fold (log2FC = −1.27) in line APL01. In addition, six genes regulating the protein ubiquitination necessary for the normal function of transcription factors^43^,^44^ showed a more than 1.8-fold reduction in line APL01, in which the putative rapeseed *UNUSUAL FLORAL ORGANS* (*UFO*) (*BnaC08g09370D*), as a *LFY* transcriptional co-factor, was down-regulated four fold (log2 = −2.01) in line APL01. Moreover, two protein farnesylation-related genes limiting the over-proliferation of meristematic cells, the putative rapeseed *PLURIPETALA* (*PLP*) (*BnaA01g18430D*) and *WIGGUM* (*WIG*) (*BnaA04g10140D* were elevated 1.8-fold (log2FC = 0.81) and two fold (log2FC = 0.99), respectively, in line APL01 compared with line PL01. This probably restricted the normal initiation of the petal primordia in line APL01. In addition, a few of floral regulatory genes whose expression changes have nonlinear relationships with the phenotypic variations in line APL01 were found as well, such as the putative rapeseed *TEMPRANILLO 2* (*TEM2*) (*BnaAnng40580D*) (log2FC = 1.02) and *PETAL LOSS* (*PTL*) (*BnaA03g01020D*) (log2FC = 0.92). Finally, based on the present study, the aforementioned 36 genes, as the upstream regulators of genes required for the basic cellular processes responsible for petal morphogenesis, may participate in the regulation of petal development in some coordinated way in *B. napus*.

### Detection of candidate genes regulating petal origination in *B. napus*

**Figure 6:**
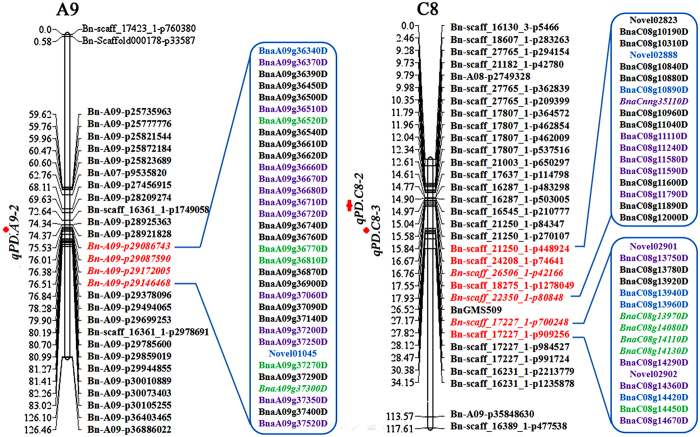
Identification of candidate genes regulating petal origination. The linkage groups are represented by vertical bars. Red loci underlie the CIs of the three QTLs, *qPD.A9-2*, *qPD.C8-2* and *qPD.C8-3*. Italicized loci are distinguishable between lines APL01 and PL01. DEGs in the blue boxes underlie the CIs of the three QTLs, in which blue genes are near the valid SNPs identified by 60 K Infinium BeadChip Array and RNA-seq, violet genes have the same dynamic expression levels between lines APL01 and PL01 as between APL01 and ’Holly’, and green genes are both. Italicized genes represent the potential candidate genes.

To further confirm the regulators of the apetalous characteristic in line APL01, three steady QTLs for PDgr were identified in the population, termed ‘AH’, containing 189 individuals derived from a cross between line APL01 and the normally petalled variety ‘Holly’ in our previous study, and designated as *qPD.A9-2*, *qPD.C8-2* and *qPD.C8-3*^34^ (Fig. 6). There are four, five and two single nucleotide polymorphisms (SNPs) in the confidence intervals (CIs) of the three QTLs^34^ (Fig. 6). Based on the comparative mapping between the ‘AH’ map and the *B. napus* genome, 223, 266 and 110 genes underlying the CIs of *qPD.A9-2*, *qPD.C8-2* and *qPD.C8-3*, respectively, were obtained.

In this study, 13,205 DEGs were identified in the young inflorescences of line APL01 when compared with those of line PL01. Underlying the CIs of the three QTLs of PDgr, 33, 18 and 16 DEGs were obtained (Fig. 6, Supplementary Table S5). Subsequently, the expression patterns of these DEGs were analyzed between APL01 and ‘Holly’ by qRT-PCR. The expression patterns of the 34 DEGs were similar to the RNA-seq assay results (Fig. 6, Supplementary Table S5).

Furthermore, 11 SNPs underlying the CIs of the three QTLs were verified between lines APL01 and PL01. As shown in Fig. 6, seven SNPs, *Bn-A09-p29086743*, *Bn-A09-p29087590*, *Bn-A09-p29172005*, *Bn-A09-p29146468*, *Bn-scaff_26506_1-p42166*, *Bn-scaff_22350_1-p80848* and *Bn-scaff_17227_1-p700248*, were distinguishable between lines APL01 and PL01. In combination with the 71 SNPs detected in RNA-seq assay, as well as located in the CIs of the three QTLs (Supplementary Data 5), 17 genes near these SNPs were obtained, of which 10 genes (indicated in green) showed the same expression changes between lines APL01 and PL01 as between APL01 and ‘Holly’. In the end, six genes (indicated in italics) were considered as the potential candidate genes regulating the petal development of line APL01. Homology analysis showed that these genes were possibly involved in protein transport, branched-chain amino acid metabolic process, the control of gene transcription, respectively (Table 3). In a future study, genetic transformation methods will be used to determine the functions of these genes.

## Discussion

In the present study, line PL01 was more resistant to bolting than line APL01 under the same vernalization and light conditions, implying that the differences occurred in at least the vernalization and photoperiod pathways. In the meantime, the early flower development of line PL01 is similar to that in *Arabidopsis*, except that the initiation of the petal primordial occurs later than that of the stamen primordia^38^, which is consistent with a previous study in *B. napus*^45^. Unlike line PL01, the petal primordial of line APL01 do not appear in the second whorl (Fig. 1C), suggesting that the apetalous character of line APL01 is formed at the initial petal primordia stage. Moreover, the remaining floral organs of line APL01 are fully developed, which distinguishes the line from apetalous mutants of *Arabidopsis* and *Antirrhinum* with variant sepals or stamens^7^,^8^, leading to the speculation that the regulation of genes that downstream ABC class genes in petal development pathways might have been changed^12^.

Subsequently, RNA-seq assays revealed that a large number of genes responsible for protein biosynthesis were down-regulated, while a large number of genes competitively impeding protein biosynthesis were up-regulated in line APL01. This may correspond to the variation in petal origination in line APL01, because protein biosynthesis as a vital component of the basic cellular processes responsible for cell proliferation and differentiation is required for the formation of petal primordia^46^,^47^. Meanwhile, many of genes up-regulated in line APL01 were aggregated in the category of “acid-amino acid ligase activity”, which promotes protein biosynthesis and is possibly responsible for the early flowering of line APL01. Likewise, the fact that protein biosynthesis acts as a vital component of the basic cellular processes responsible for the formation of the FMs has been confirmed in *Arabidopsis*^48^,^49^.

Furthermore, a large number of homologs of the *Arabidopsis* floral regulatory genes were identified in the *B. napus* genome through homologous alignment. However, a portion of these genes was not expressed in the lines APL01 and PL01, which might be due to the psuedolization or neofunctionalization^19^,^50^. Of the 1093 putative rapeseed flowering genes, 82 DEGs possibly participated in the regulation of early flowering in line APL01. These DEGs are involved in multiple flowering pathways, such as vernalization, photoperiod and GA. This indicates that the floral transition is indeed complicated^1^,^2^. However, a further analysis indicated that most of the genes involved in vernalization and photoperiod pathways, such as the down-regulated *FLC* (log2FC = −1.94) and the up-regulated *CO* (log2FC = 3.11), displayed more than two fold changes in gene expression levels between lines APL01 and PL01, suggesting that the early flowering of line APL01 was predominantly attributed to the vernalization and photoperiod pathways (Fig. 5), which is consistent with the phenotypic analyses. In addition, only a few genes functioning in each pathway showed differential expression levels in lines APL01 and PL01, implying that these genes probably regulate the early flowering of line APL01 as well (Fig. 5), but this needs to be confirmed through additional phenotypic studies. Eventually, the up-regulated FM-associated genes, *LFY* (log2FC = 0.93) and *CAL* (log2FC = 2.08), promoted faster floral transitions from SAM to FM in line APL01 compared with line PL01 probably by up-regulating a number of genes implicated in “acid-amino acid ligase activity”^48^,^49^.

Among the 372 putative rapeseed petal regulators, *AP1*, *AP3* and *PI* showed no obvious changes in gene expression levels between lines APL01 and PL01, implying that the downstream regulators of B-class genes, or an unknown regulatory network, govern the apetalous characteristic of line APL01. The 36 upstream genes involved in petal development probably give rise to the apetalous characteristic of line APL01 by down-regulating the genes responsible for protein biosynthesis and/or by up-regulating the genes that competitively inhibit protein biosynthesis. However, the regulatory mechanism is highly evolved and obviously different from *Arabidopsis* in *B. napus*, because the apetalous mutants of the 36 genes, such as *AIL5*, *PNY* and *TSL*, in *Arabidopsis* are invariably accompanied by abnormal sepals or/and stamens^17^,^18^,^51^. For the up-regulated *TEM2* and *PTL* in line APL01, one plausible explanation is that those genes acquire novel functions because of the frequent segmental duplications and polyploidization events^52^. Alternatively, the disruption of certain regulatory elements in the promoters of the duplicated genes may lead to altered expression patterns and hence to sub-functionalization^52^.

Floral transition and petal morphogenesis, as the two main components of flower development, are tight related to each other. In *Arabidopsis*, the 32 flowering time genes also work to regulate petal development, of which 19 genes have identical effects, while 13 genes have inverse effects on floral transition as in petal morphogenesis (Supplementary Data 4). In this study, the putative rapeseed *MULTICOPY SUPRESSOR OF IRA1* (*MSI1*) (*BnaA03g09860D*) (log2FC = 2.25), *PNY* (*BnaAnng29380D*) (log2FC = −0.88) and (*BnaC03g00520D*) (log2FC = −∞), and *TSL* (*BnaA10g14620D*) (logFC = −1.92), which had expression changes that were consistent with the phenotypic changes between lines APL01 and PL01, probably govern early flowering as well as the apetalous characteristic in line APL01^18^,^51^,^53^. Interestingly, two putative rapeseed *EMBRYONIC FLOWER 1* (*EMF1*) genes (*BnaA03g03410D* and *BnaC03g04840D*), repressors of floral transition and petal development^54^, respectively, showed 118.7-fold (log2FC = −6.89) down-regulation and 6.33-fold (log2FC = 2.66) up-regulation in line APL01, indicating that the down-regulated gene may be specifically responsible for the regulation of floral transition, while the up-regulated gene specifically regulates petal development in line APL01. This phenomenon probably occurs universally in polyploids because of polyploidization and functional differentiation^52^.

The excavation of candidate genes that act as important components of this study is crucial to explain the molecular mechanisms controlling petal origination in *B. napus*. Based on the comparative mapping between the ‘AH’ map and the *B. napus* genome, 328 genes underlying the CIs of three QTLs regulating PDgr were obtained in our previous study^34^. In the present study, the comparison of An-Ar and Cn-Co for orthologous gene pairs underlying the CIs of the three QTLs was performed because the *B. napus* An and Cn subgenomes are largely colinear to the corresponding diploid Ar (*B. rapa*) and Cn (*B. oleracea*) genomes^19^. Finally, 599 genes underlying the CIs of the three QTLs were obtained. Curiously, these genes contain none of the 372 homologs functioning in petal development in *B. napus*, which may be attributed to novel regulators controlling petal development. In combination with the expression data from RNA-seq and qRT-PCR assays, 10 genes showed the same dynamic expression levels between lines APL01 and PL01 as between APL01 and ‘Holly’ (Fig. 6), implying that these genes were possibly implicated in petal development. In combination with the SNPs closely associated to the apetalous characteristic, six genes were considered as potential candidate genes for regulating the PDgr of oilseed rape (Fig. 6). Together with the previous study^55^, these findings suggested that RNA-seq in association with QTL mapping might be a feasible manner to detect target genes governing PDgr and even other quantitative traits.

## Methods

### Plant materials

*B. napus* lines APL01 and PL01, an absolutely apetalous variety and a normally petalled variety, respectively, were derived from the F_6_ generation of crosses between apetalous (‘Apetalous No. 1’) and normal petalous (‘Zhongshuang No. 4’) oilseed rape in 1998. ‘Apetalous No. 1’ was bred from the F_8_ generation of crosses between China oilseed rape cultivar with smaller petals (SP103) and *B. rapa* variety with lower petals (LP153). ‘Zhongshuang No. 4’ was developed at the Oil Crops Research Institute of the Chinese Academy of Agricultural Sciences, Wuhan, China. Most of traits are similar between lines APL01 and PL01, except for early flowering and apetalous characteristics. Seeds of lines APL01 and PL01 were sown in the field in October 2014, and then at least 30 plants for each line were used to count the number of rosette leaves and days of vegetative growth at the initial flowering stage. In the meantime, after a 4 °C treatment for 50 d, seeds were sown in a growth chamber. The light intensity was 8000 Lux for a 16 h daily light period, and day and night temperatures were 25 and 15 °C, respectively. Plants were watered every few days. All 20 plants for each line were used to count the numbers of rosette leaves and flowering plants, and days of vegetative growth at the beginning flower stage. Lines APL01 and PL01 that were planted in the field or greenhouse were always grown in a common condition. Non-paired *t*-test, SPSS Statistics 19.0 software and Sigma Plot 12.5 software were used to create Fig. 1A,B.

### Paraffin section assay

Young buds at stages 3 to 12 were collected from lines APL01 and PL01. Bud samples were fixed in FAA (45% absolute ethanol, 5% glacial acetic acid, 5% formaldehyde and 45% ddH_2_O) buffer for 24 h, then dehydrated through an ethanol series and embedded in paraffin. Finally, 7 μm sections were generated using a Leica RM2235 (Leica, Wetzlar, Germany) and stained with 1% fast green.

### Total RNA extraction and quality test

Young inflorescences that only consist of inflorescence shoot apical meristem and buds at stages 1 to 5 were collected from lines APL01 and PL01, and three biological replicates were performed for each line. Then, total RNA was isolated using MagaZorb^®^ Total RNA Mini-Prep Kit (Promega, Madison, USA). RNA degradation and contamination were monitored on 1% agarose gels. RNA purity was checked using the NanoPhotometer^®^ spectrophotometer (IMPLEN, CA, USA). RNA concentration was measured using a Qubit RNA Assay Kit on a Qubit 2.0 Fluorometer (Life Technologies, CA, USA). RNA integrity was assessed using the RNA Nano 6000 Assay Kit of the Bioanalyzer 2100 system (Agilent Technologies, CA, USA). Only pure RNA samples of high quality were used in RNA-seq.

### RNA-seq library construction and sequencing

Total six sequencing libraries were generated using NEBNext Ultra RNA Library Prep Kit for Illumina (NEB, USA) following the manufacturer’s recommendations, and six index codes were added to attribute the sequences to the appropriate sample. Library quality was assessed on the Agilent Bioanalyzer 2100 system. The clustering of the index-coded samples was performed on a cBot Cluster Generation System using TruSeq PE Cluster Kit v3-cBot-HS (Illumina) according to the manufacturer’s instructions. After cluster generation, the library preparations were sequenced on an Illumina Hiseq 2500 platform, and 125 bp paired-end reads were generated.

### RNA-seq data analysis

Raw reads in the fastq format were firstly processed using in-house Perl scripts. Clean reads of high quality were obtained by removing reads containing adapters, reads containing ‘ploy’ Ns and low quality reads from the raw data. Paired-end clean reads were aligned to the *B. napus* genome (http://www.genoscope.cns.fr/brassicanapus/) using TopHat^19^,^39^. Subsequently, HTSeq was used to count the read numbers mapped to each gene. Then, the RPKM of each gene was calculated based on the length of the gene, and the reads count mapped to this gene^56^. Further, the Pearson correlation coefficients between six samples were generated using the R programming language (Fig. 2A, Supplementary Fig. S2)^57^. A differential expression analysis of two groups (three biological replicates per group) was performed using the DESeq R package. The resulting *P* values were adjusted using the Benjamini and Hochberg’s approach for controlling the false discovery rate (Supplementary Data 1)^58^. Genes with an adjusted *P* value < 0.05, as well as at least a 1.6-fold change in gene expression, were assigned as differentially expressed (Fig. 2C).

### GO enrichment analysis

A GO enrichment analysis of the DEGs was implemented using the GOseq R package^42^, in which gene length bias was corrected. GO terms with corrected *P* values less than 0.05 were considered significantly enriched by DEGs (Fig. 4). For each of the three kinds of GO terms that were significantly enriched, a DAG was drawn using the topGO R package to illuminate the relationships among significantly enriched GO terms (Supplementary Fig. S3)^59^.

### Quantitative real-time RT-PCR assay

For young inflorescences independently collected from APL01, PL01 and Hollyat the same developmental stages as used for the RNA-seq analysis, total RNA extraction and purification were performed as described above, and three biological replicates were performed for each line. cDNAs were biosynthesized using the PrimeScript RT reagent kit (TaKaRa, Da Lian, China). Primers for qRT-PCR were designed using the Primer 5 software (http://www.premierbiosoft.com/primerdesign/) and synthesized by Sangon Biotech. The gene-specific primers are listed in Supplementary Table S6. The rapeseed *ACTIN* gene was used as an internal control, and triplicate quantitative assays were performed on each cDNA dilution using the SYBR^®^ Premix Ex Taq™ (Tli RNaseH Plus) (Takara) with the ABI PRISM 7500 Real-Time PCR System (Applied Biosystems, USA). The relative expression level of each gene was calculated using the 2^−ΔΔCt^ method^60^ and the standard deviation was calculated from three biological replicates (Supplementary Fig. S4, Supplementary Table S1 and S5). The Pearson correlation coefficients between RNA-seq data and qRT-PCR data was calculated using SPSS Statistics 19.0 software, and Sigma Plot 12.5 software was used to create Fig. 3.

### RNA-seq associated with QTL mapping

In our previous study, the ‘AH’ population, a recombinant inbred line containing 189 individuals, was generated from a cross between APL01 and a normally petalled variety ‘Holly’^34^. Then, three major QTLs associated with PDgr were detected^34^. On the basis of the comparative mapping between the ‘AH’ map and the *B. napus* reference genome, genes underlying the CIs of the three QTLs were obtained^34^.

However, high-throughput RNA-seq was performed with young inflorescences independently collected from lines APL01 and PL01. For the genes differentially expressed in the line APL01 when compared with line PL01, those DEGs located in the CIs of the three QTLs were verified in young inflorescences independently generated from APL01 and ‘Holly’ at the same developmental stage by qRT-PCR. DEGs whose expression patterns were similar in both assays were used.

Further, the validity of the SNPs located in the CIs of the three QTLs was verified between lines APL01 and PL01. At the same time, in combination with SNPs identified in the RNA-seq assay using GATK2 software, DEGs that were screened in the previous step and were located near valid SNPs, were considered as potential candidate genes regulating PDgr in *B. napus*.

## Additional Information

**How to cite this article**: Yu, K. *et al*. Genome-wide transcriptomic analysis uncovers the molecular basis underlying early flowering and apetalous characteristic in *Brassica napus* L. *Sci. Rep.* **6**, 30576; doi: 10.1038/srep30576 (2016).

## Supplementary Material

Supplementary Information

Supplementary Data 1

Supplementary Data 2

Supplementary Data 3

Supplementary Data 4

Supplementary Data 5

## Figures and Tables

**Table 1:** Summary of transcriptome sequencing data.

Sample name	Raw reads	Clean reads (Clean/All)	Total mapped (Mapped/Clean)	Uniquely mapped (Uniquely/Clean)
APL01_1	65402962	64459220 (98.56%)	53608254 (83.17%)	50571809 (78.46%)
APL01_2	69377420	68424392 (98.63%)	56185999 (82.11%)	52962825 (77.4%)
APL01_3	60303396	59493724 (98.66%)	49378266 (83%)	46560393 (78.26%)
PL01_1	56009894	55312424 (98.75%)	45866936 (82.92%)	43351994 (78.38%)
PL01_2	57018874	56255752 (98.66%)	46676182 (82.97%)	44095732 (78.38%)
PL01_3	64562006	63753424 (98.75%)	52898180 (82.97%)	50025495 (78.47%)

**Table 2:** List of DEGs that impeded petal development in line APL01.

Gene id	Arabidopsis homologue	Gene name	Function description	Function	Log2FoldChange	padj
BnaA06g14090D	AT5G62000	ARF2	transcription factor	Repressor	+∞	2.76E-64
BnaA05g14370D	AT5G62000	ARF2	transcription factor	Repressor	10.293	2.34E-59
BnaA07g25390D	AT2G33860	ARF3	transcription factor	Repressor	0.80589	0.022004
BnaAnng29380D	AT5G02030	PNY	transcription factor	Activator	−0.87659	0.013344
BnaA10g13520D	AT5G60690	IFL	transcription factor	Activator	−0.91016	0.010943
BnaCnng73930D	AT5G57390	AIL5	transcription factor	Activator	−0.91498	0.041957
BnaA10g18480D	AT5G15800	SEP1	transcription factor	Activator	−1.0138	0.0029356
BnaA07g38010D	AT2G45190	FIL	transcription factor	Activator	−1.4698	1.80E-05
BnaC05g10940D	AT1G14760	KNATM	transcription factor	Activator	−1.4822	0.036086
BnaC07g11300D	AT1G24260	SEP3	transcription factor	Activator	−1.725	0.003165
BnaC03g01370D	AT5G03680	PTL	transcription factor	Activator	−2.0615	3.63E-05
BnaA06g09570D	AT1G14760	KNATM	transcription factor	Activator	−2.846	0.0053783
BnaC01g36350D	AT3G15170	CUC1	transcription factor	Activator	−2.9968	0.0034186
BnaC09g49350D	AT5G06070	RBE	transcription factor	Activator	−4.4325	0.017223
BnaC03g00520D	AT5G02030	PNY	transcription factor	Activator	−∞	6.93E-05
BnaC05g48320D	AT3G02310	SEP2	transcription factor	Activator	−∞	1.60E-83
BnaA10g11890D	AT5G37055	SEF	epigenetic regulation	Repressor	6.9775	1.03E-21
BnaA03g03410D	AT5G11530	EMF1	epigenetic regulation	Repressor	2.6625	1.47E-14
BnaA03g09860D	AT5G58230	MSI1	epigenetic regulation	Repressor	2.2538	1.68E-13
BnaCnng01170D	AT4G02020	SWN	epigenetic regulation	Repressor	1.6771	0.00094501
BnaAnng03220D	AT3G06400	CHR11	epigenetic regulation	Repressor	1.6757	5.85E-08
BnaA08g00100D	AT3G33520	ARP6	epigenetic regulation	Repressor	0.98694	0.0049042
BnaA06g16550D	AT3G48430	JMJ12	epigenetic regulation	Activator	−1.0094	0.015536
BnaC02g13170D	AT5G55300	TOP1ALPHA	epigenetic regulation	Activator	−1.5772	1.41E-05
BnaA10g14620D	AT5G20930	TSL	epigenetic regulation	Activator	−1.9186	3.60E-09
BnaC09g21160D	AT2G03070	MED8	transcriptional co-activator	Activator	−1.2735	0.00034295
BnaA02g12180D	AT1G65620	AS2	transcriptional repressor	Activator	−1.3106	0.027009
BnaA07g19900D	AT1G15750	TPL	transcriptional repressor	Repressor	1.5076	1.48E-06
BnaC04g12390D	AT2G32410	AXL1	protein ubiquitination	Activator	−0.84903	0.048187
BnaA07g32230D	AT5G42190	ASK2	protein ubiquitination	Activator	−1.031	0.044645
BnaC05g15660D	AT2G25700	ASK3	protein ubiquitination	Activator	−1.0949	0.023784
BnaA09g50410D	AT1G05180	AXR1	protein ubiquitination	Activator	−1.3034	0.0061456
BnaA02g17620D	AT1G75950	ASK1	protein ubiquitination	Activator	−1.6267	3.00E-07
BnaC08g09370D	AT1G30950	UFO	protein ubiquitination	Activator	−2.0059	8.83E-05
BnaA04g10140D	AT5G40280	WIG	protein farnesylation	Repressor	0.99166	0.010723
BnaA01g18430D	AT3G59380	PLP	protein farnesylation	Repressor	0.81325	0.026371

“Activator” indicates that gene positively regulates petal development.

“Repressor” indicates that gene negatively regulates petal development.

“+∞” indicates that gene is only expressed in line APL01.

“−∞” indicates that gene is only expressed in line PL01.

**Table 3:** Candidate genes identified within the CIs of *qPD.A9-2*, *qPD.C8-2* and *qPD.C8-3*.

*B. napus* gene	*A. thaliana* homolog	*A. thaliana* function description
BnaA09g37300D	AT3G58170	Bet1/Sft1-like SNARE protein; Involved in ER to Golgi vesicle-mediated protein transport
BnaCnng35110D	AT3G43790	Zinc induced facilitator-like 2 (ZIFL2); Carbohydrate transmembrane transporter activity
BnaC08g13970D	AT1G10070	Chloroplast branched-chain amino acid aminotransferase; Involved in branched-chain amino acid metabolic process Branched-chain-amino-acid transaminase activity
BnaC08g14080D	—	—
BnaC08g14110D	AT1G10200	LIM proteins; Involved in actin filament bundle assembly; Zinc ion binding; Transcription factor activity
BnaC08g14130D	AT1G10270	Glutamine-rich protein 23 (GRP23); Involved in cell division and regulation of transcription; Protein binding activity
