# Supplementary material for: Genome-wide transcriptomic analysis uncovers the molecular basis underlying early flowering and apetalous characteristic in Brassica napus L
Source: Sci Rep. 2016 Jul 27;6:30576. doi: 10.1038/srep30576 (PMC4962316; doi:10.1038/srep30576)
Supplement: Supplementary Information [file srep30576-s1.pdf]

Genome-wide transcriptomic analysis uncovers the molecular basis underlying early flowering and apetalous characteristic in *Brassica napus* L.

Kunjiang Yu[1,2†], Xiaodong Wang[2†], Feng Chen[2], Song Chen[2], Qi Peng[2], Hongge Li[2], Wei Zhang[2], Maolong Hu[2], Pu Chu[1], Jiefu Zhang[2*], Rongzhan Guan[1*]

[1]State Key Laboratory of Crop Genetics and Germplasm Enhancement, Nanjing Agricultural University, Nanjing, 210095, China

[2]Key Laboratory of Cotton and Rapeseed, Ministry of Agriculture/Institute of Industrial Crops, Jiangsu Academy of Agricultural Sciences, Nanjing, 210014, China

[†]These authors contributed equally to this work.

[*]Corresponding authors contributed equally to this work. jiefu_z@163.com; guanrzh@njau.edu.cn.

SUPPLEMENTARY FIGURES

Supplementary Figure S1. Comparison of flowering time and petal morphology between lines APL01 and PL01.

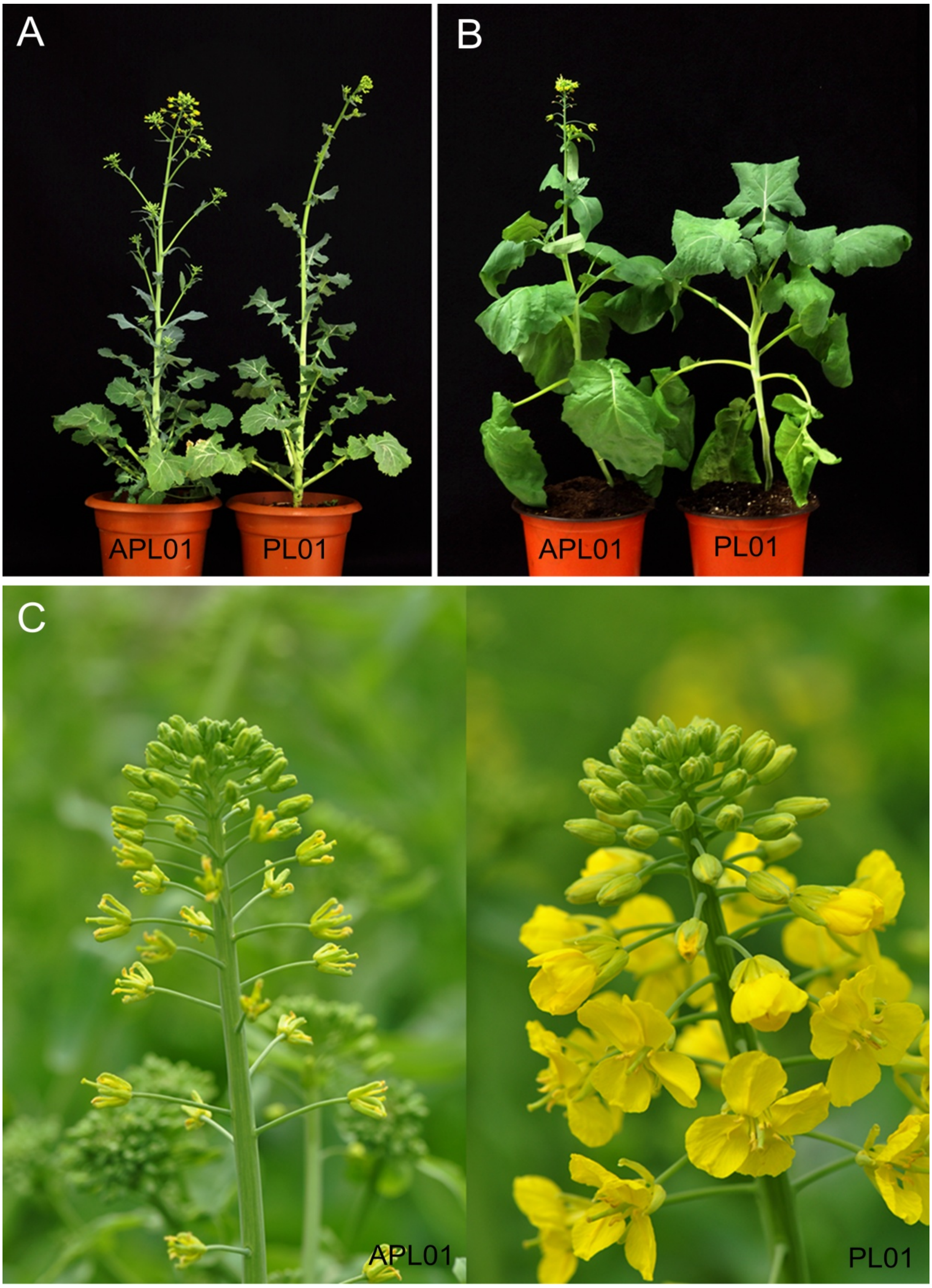

(S1A) Line PL01 is bolting and budding when line APL01 began to put forth their blossoms in the field. (S1B) Line PL01 is still at vegetative growth stage when line APL01 began to flower in the greenhouse. (S1C) Line APL01 is absolutely apetalous while line PL01 is normally petalous.

Supplementary Figure S2. Correlation between genes expression levels of three biological replicates for each line.

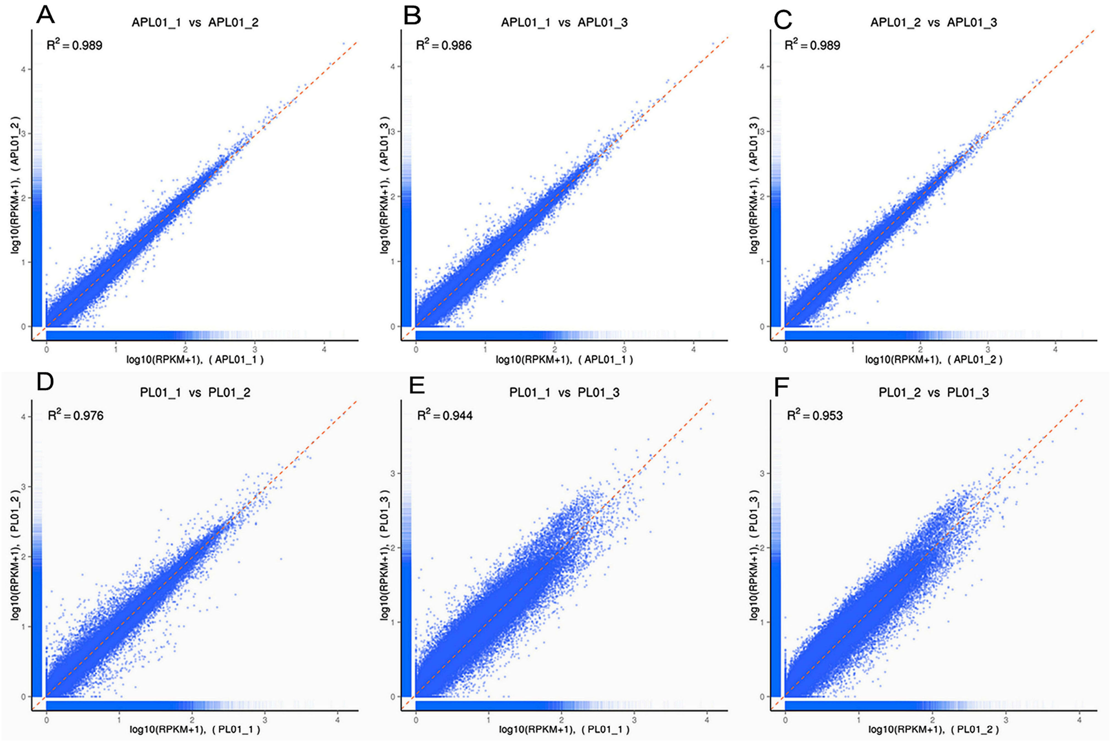

(S2A) Pearson Correlation Coefficient between genes expression levels of APL01_1 and APL01_2. (S2B) Pearson Correlation Coefficient between genes expression levels of APL01_1 and APL01_3. (S2C) Pearson Correlation Coefficient between genes expression levels of APL01_2 and APL01_3. (S2D) Pearson Correlation Coefficient between genes expression levels of PL01_1 and PL01_2. (S2E) Pearson Correlation Coefficient between genes expression levels of PL01_1 and PL01_3. (S2F) Pearson Correlation Coefficient between genes expression levels of PL01_2 and PL01_3. R^2>0.8 as the significance cutoffs.

Supplementary Figure S3. Directed acyclic graph (DAG) of GO terms (corrected *P* value<0.05) significantly enriched by down-regulated or up-regulated genes.

A

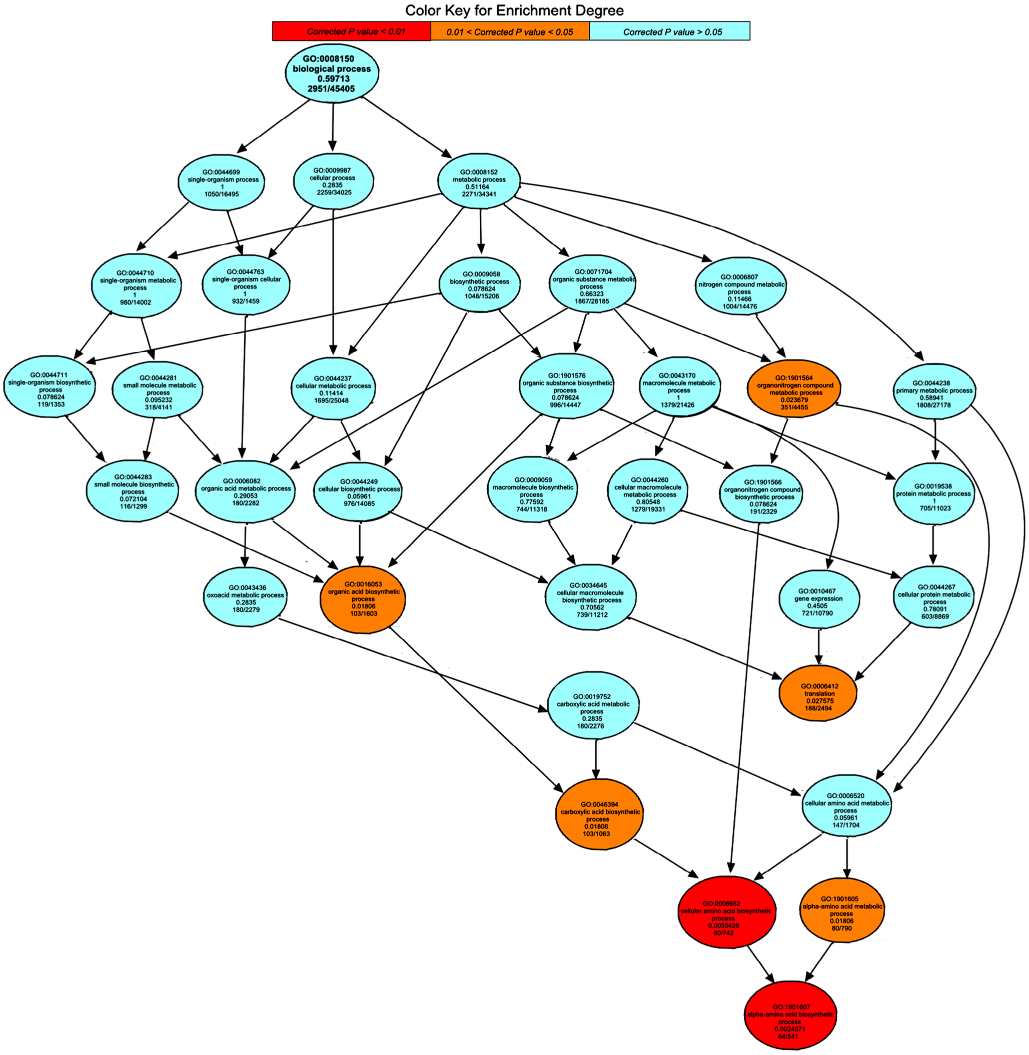

(S3A) The DAG of GO terms significantly enriched by down-regulated genes in the biological process group. Corrected *P* value<0.05 as the significance cutoffs.

B

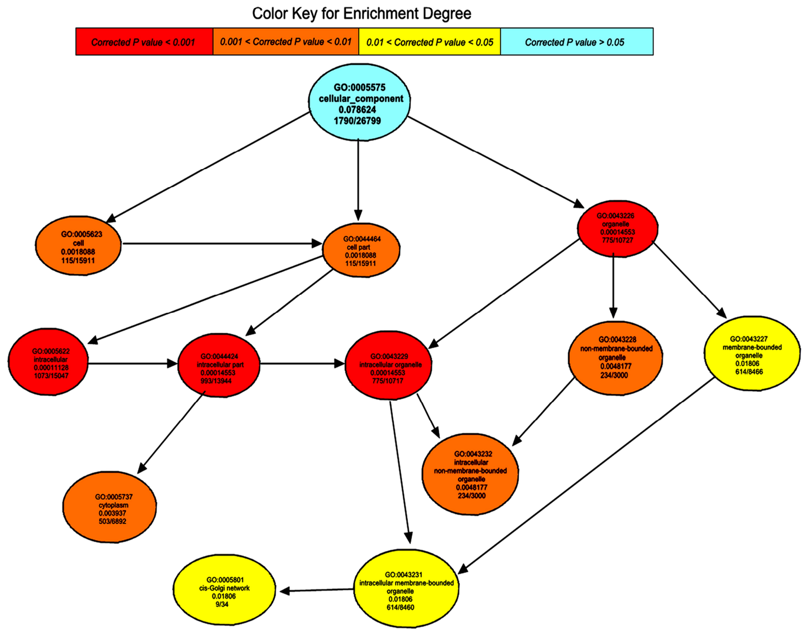

(S3B) The DAG of GO terms significantly enriched by down-regulated genes in the cellular component group. Corrected *P* value<0.05 as the significance cutoffs.

C

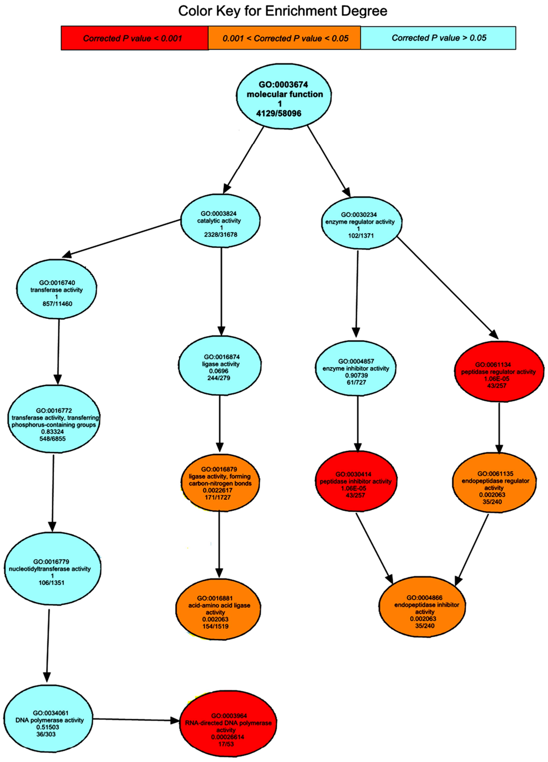

(S3C) The DAG of GO terms significantly enriched by up-regulated genes in the molecular function group. Corrected *P* value<0.05 as the significance cutoffs.

Supplementary Figure S4. Relative expression of 18 photoperiod-related genes in the young leaves of line APL01 and line PL01.

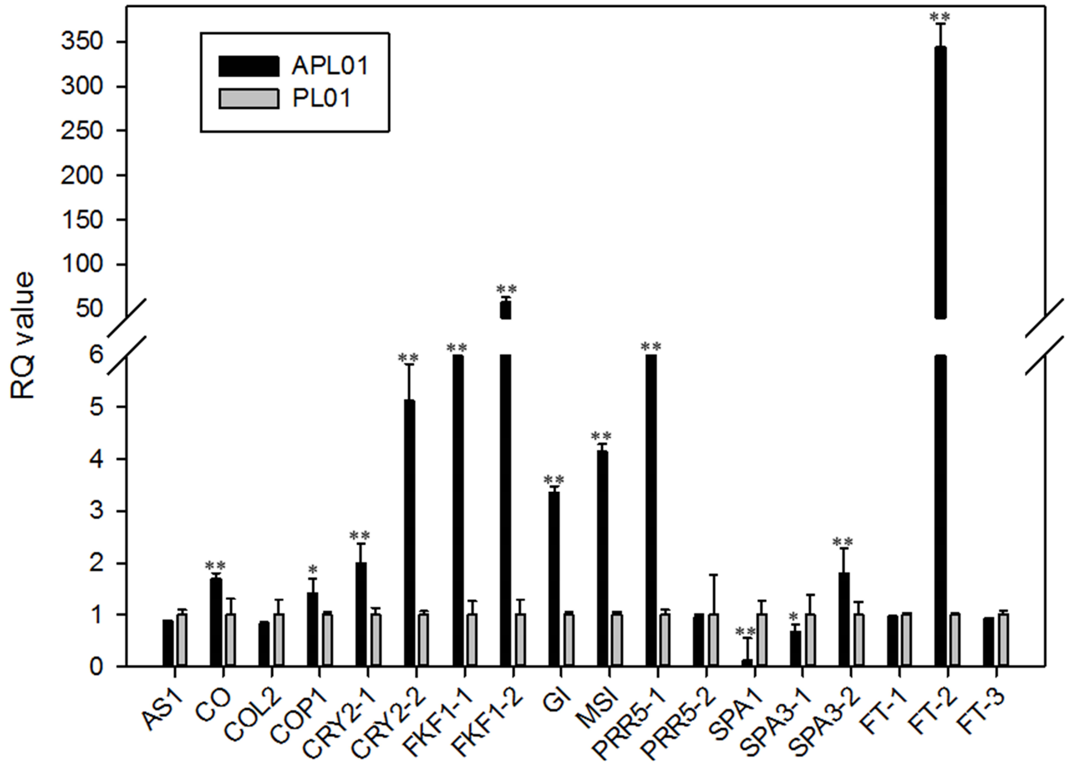

The expression patterns of three *FT* genes and 15 photoperiod-related DEGs identified by RNA-seq were verified in young leaves from lines APL01 and PL01 through qRT-PCR. RQ value represents the relative expression level of each gene. *AS1*, *BnaA05g06890D*; *CO*, *BnaA02g02840D*; *COL2*, *BnaC03g32910D*; *COP1*, *BnaA05g34990D*; *CRY2-1*, *BnaA08g27870D*; *CRY2-2*, *BnaA10g02550D*; *FKF1-1*, *BnaA07g01250D*; *FKF1-2*, *BnaC07g02620D*; *GI*, *BnaA09g30390D*; *MSI1*, *BnaA03g09860D*; *PRR5-1*, *BnaA02g32550D*; *PRR5-2*, *BnaC09g04380D*; *SPA1*, *BnaA03g21350D*; *SPA3-1*, *BnaA05g24250D*; *SPA3-2*, *BnaC01g36140D*; *FT-1*, *BnaC02g23820D*; *FT-2*, *BnaA02g12130D*; *FT-3*, *BnaA07g33120D*.

SUPPLEMENTARY TABLES

Supplementary Table S1. List of DEGs verified by qRT-PCR.

Gene name	Gene id	Log2FoldChange	
		RNA-seq	qRT-PCR
ARF2	BnaA05g14370D	10.293	13.894
COL2	BnaC03g32910D	4.7	6.886
CO	BnaA02g02840D	3.1109	4.65
PRR5-2	BnaC09g04380D	3.1796	3.752
EMF1	BnaA03g03410D	2.6625	3.3245
MSI	BnaA03g09860D	2.2538	3.015
PRR5-1	BnaA02g32550D	1.3287	2.58
CRY2-1	BnaA08g27870D	0.98117	1.94
FKF1-1	BnaA07g01250D	0.8065	1.875
CRY2-2	BnaA10g02550D	0.81612	1.852
AS1	BnaA05g06890D	0.92736	1.852
ARP6	BnaA08g00100D	0.98694	1.711
CHR11	BnaAnng03220D	1.6757	0.813
FKF1-2	BnaC07g02620D	1.2422	0.73
PHYB	BnaC03g39830D	0.80852	0.6522
GI	BnaA09g30390D	1.1684	0.623
ARF3	BnaA07g25390D	0.80589	0.6115
CUC1	BnaC01g36350D	-2.9968	-3.731
ASK1	BnaA02g17620D	-1.6267	-2.512
PFT1	BnaA08g19480D	-1.2185	-2.286
ASK2	BnaA07g32230D	-1.031	-1.92
ASK3	BnaC05g15660D	-1.0949	-1.832
COP1	BnaA05g34990D	-0.79776	-1.353
AS2	BnaA02g12180D	-1.3106	-0.872
AIL5	BnaCnng73930D	-0.91498	-0.83
AXR1	BnaA09g50410D	-1.3034	-0.802
AXL1	BnaC04g12390D	-0.84903	-0.623

The expression patterns of 27 DEGs identified in the RNA-seq assays were verified in young inflorescences independently collected from lines APL01 and PL01 by qRT-PCR.

Supplementary Table S2. List of DEGs that promote early flowering in line APL01.

Gene id	Arabidopsis homologue	Gene name	Pathway	Function	log2FoldChange	padj
BnaC01g33680D	AT3G18990	VRN1	Vernalization	Activator	5.6283	5.57E-44
BnaA01g12700D	AT4G22950	AGL19	Vernalization	Activator	2.5669	0.00082241
BnaA03g35020D	AT3G18990	VRN1	Vernalization	Activator	2.5253	3.65E-11
BnaA02g08140D	AT5G57380	VIN3	Vernalization	Activator	2.5109	4.75E-14
BnaA08g10540D	AT4G22950	AGL19	Vernalization	Activator	1.5026	0.00059654
BnaA03g10310D	AT5G57380	VIN3	Vernalization	Activator	1.077	0.0075531
BnaC09g46540D	AT5G10140	FLC	Vernalization	Repressor	-1.9367	0.0094857
BnaC03g04170D	AT5G65050	MAF2	Vernalization	Repressor	-∞	2.41E-13
BnaC03g32910D	AT3G02380	COL2	Photoperiod	Activator	4.7	4.07E-05
BnaC09g04380D	AT5G24470	PRR5	Photoperiod	Activator	3.1796	0.00069593
BnaA02g02840D	AT5G15840	CO	Photoperiod	Activator	3.1109	0.023015
BnaA03g09860D	AT5G58230	MSI1	Photoperiod	Activator	2.2538	1.68E-13
BnaA02g32550D	AT5G24470	PRR5	Photoperiod	Activator	1.3287	6.58E-05
BnaC07g02620D	AT1G68050	FKF1	Photoperiod	Activator	1.2422	0.0003441
BnaA09g30390D	AT1G22770	GI	Photoperiod	Activator	1.1684	0.036052
BnaA08g27870D	AT1G04400	CRY2	Photoperiod	Activator	0.98117	0.0020872
BnaA05g06890D	AT2G37630	AS1	Photoperiod	Activator	0.92736	0.0095852
BnaA10g02550D	AT1G04400	CRY2	Photoperiod	Activator	0.81612	0.025342
BnaA07g01250D	AT1G68050	FKF1	Photoperiod	Activator	0.8065	0.035115
BnaA05g34990D	AT2G32950	COP1	Photoperiod	Repressor	-0.79776	0.030493
BnaA05g24250D	AT3G15354	SPA3	Photoperiod	Repressor	-0.85161	0.047493
BnaA03g21350D	AT2G46340	SPA1	Photoperiod	Repressor	-∞	3.28E-24
BnaC01g36140D	AT3G15354	SPA3	Photoperiod	Repressor	-∞	9.50E-07
BnaCnng55170D	AT3G63010	GID1B	GA	Activator	1.0407	0.0013179
BnaA09g20650D	AT5G27320	GID1C	GA	Activator	0.91979	0.0047467
BnaC05g46680D	AT3G05120	GID1A	GA	Activator	0.88106	0.010614
BnaC09g05150D	AT5G61060	HDA5	Autonomous	Activator	+∞	0.0095921
BnaC01g44770D	AT3G13682	LDL2	Autonomous	Activator	4.4007	3.72E-27
BnaA02g20680D	AT4G02560	LD	Autonomous	Activator	1.6503	8.67E-07
BnaA05g09840D	AT2G33810	SPL3	Aging	Activator	1.9664	2.15E-09
BnaA04g24340D	AT2G42200	SPL9	Aging	Activator	0.99048	0.0062572
BnaA05g24340D	AT3G15270	SPL5	Aging	Activator	0.79657	0.027511
BnaA04g12990D	AT2G22540	SVP	Ambient temperature	Repressor	-1.3602	0.00040049
BnaCnng24550D	AT5G61850	LFY	Integrator	Activator	0.93075	0.0060388
BnaC04g50370D	AT2G45660	SOC1	Integrator	Activator	0.78644	0.049978
BnaA03g13730D	AT2G30140	UGT87A2	Unknown	Activator	2.1887	3.94E-05
BnaA03g13740D	AT2G30140	UGT87A2	Unknown	Activator	1.9784	1.31E-08
BnaC03g58740D	AT1G28520	VOZ1	Unknown	Activator	1.8932	4.54E-06
BnaC07g47240D	AT4G39400	BRI1	Unknown	Activator	0.93246	0.0061953
BnaC03g16650D	AT2G30140	UGT87A2	Unknown	Activator	0.92346	0.012513

BnaC04g48260D	AT2G42400	VOZ2	Unknown	Activator	0.92197	0.0086458
BnaA09g29300D	AT1G26830	CUL3A	Unknown	Activator	0.90249	0.0093152
BnaA06g26760D	AT5G24240	PI4Kγ3	Unknown	Repressor	-0.7912	0.03578
BnaA06g21380D	AT5G61920	FLX4	Unknown	Repressor	-0.90954	0.047261
BnaCnng05600D	AT3G01460	MBD9	Unknown	Repressor	-0.93626	0.0072842
BnaC08g39660D	AT1G14400	UBC1	Unknown	Repressor	-0.98868	0.0058431
BnaC08g38020D	AT1G17060	SOB7	Unknown	Repressor	-1.8982	0.020614
BnaC04g42250D	AT2G31650	SDG27	Unknown	Repressor	-1.9339	0.015945
BnaA08g24010D	AT1G14400	UBC1	Unknown	Repressor	-2.0603	3.77E-11
BnaC08g26470D	AT3G60870	AHL18	Unknown	Repressor	-1.0956	0.0009389
BnaA06g22130D	AT5G62640	ELF5	Unknown	Repressor	-1.1731	0.00051513
BnaC09g06200D	AT5G63980	FRY1	Unknown	Activator	+∞	7.63E-39
BnaC07g37100D	AT4G22140	EBS	Unknown	Repressor	-∞	2.84E-12
BnaA09g27250D	AT1G28420	RLT1	Unknown	Repressor	-0.80395	0.028281
BnaA02g35090D	AT5G18560	PUCHI	Unknown	Activator	6.1866	2.32E-19
BnaA07g17180D	AT3G57130	BOP1	Unknown	Activator	3.9033	3.90E-10
BnaA09g40340D	AT3G62980	TIR1	Unknown	Activator	1.0134	0.0027963
BnaA03g39820D	AT5G60910	FUL	Unknown	Activator	0.93322	0.0038222
BnaA03g31260D	AT3G10480	NAC050	Unknown	Repressor	-0.78666	0.047757
BnaA07g12050D	AT5G67180	TOE3	Unknown	Repressor	-1.889	0.0075228
BnaA07g25390D	AT5G37020	ARF8	auxin signal transduction	Activator	0.80589	0.022004
BnaC05g13090D	AT1G78580	TPS1	carbohydrate metabolism	Activator	+∞	0.039817
BnaAnng08760D	AT5G19220	ADG2	carbohydrate metabolism	Activator	3.0623	1.58E-05
BnaA03g55140D	AT5G51820	PGM1	carbohydrate metabolism	Activator	2.2655	3.15E-06
BnaCnng18320D	AT3G03090	VGT1	carbohydrate metabolism	Activator	1.564	2.14E-05
BnaC02g08620D	AT5G19220	ADG2	carbohydrate metabolism	Activator	0.79896	0.048176
BnaA10g07730D	AT5G51820	PGM1	carbohydrate metabolism	Activator	0.78116	0.035136
BnaC03g56640D	AT1G26310	CAL	Homolog of AP1	Activator	2.0825	6.92E-11
BnaA02g01960D	AT5G14010	KNU	Unknown	Activator	5.2155	0.00015447
BnaC02g05100D	AT5G14010	KNU	Unknown	Activator	2.3568	0.0010807
BnaA08g26200D	AT1G09700	HYL1	Unknown	Activator	1.8106	1.26E-06
BnaA05g30000D	AT3G07610	IBM1	Unknown	Activator	1.574	1.04E-06
BnaA04g12350D	AT2G21660	GRP7	Unknown	Activator	1.1509	0.0077118
BnaA02g24890D	AT5G47010	UPF1	Unknown	Repressor	-0.7354	0.049423
BnaA07g04040D	AT2G15790	SQN	Unknown	Repressor	-0.91632	0.017223
BnaAnng15130D	AT5G64360	EIP9	Unknown	Repressor	-1.013	0.0085354

BnaA07g20390D	AT1G79280	NUA	Unknown	Repressor	-1.0312	0.002385
BnaAnng32970D	AT3G19980	FYPP3	Unknown	Repressor	-1.3006	0.00019756
BnaC08g28320D	AT3G57390	AGL18	Unknown	Repressor	-1.552	0.0062405
BnaA02g11870D	AT1G65010	--	Unknown	Repressor	-2.1107	1.60E-10
BnaC09g19900D	AT5G47010	UPF1	Unknown	Repressor	-4.6427	1.77E-06
BnaC03g04840D	AT5G11530	EMF1	Unknown	Repressor	-6.8912	1.70E-44

"Activator" indicates that gene positively regulates floral transition.

"Repressor" indicates that gene negatively regulates floral transition.

"+∞" indicates that gene is only expressed in line APL01.

"-∞" indicates that gene is only expressed in line PL01.

Table S3. Selected references for the gene functions summarized in Figure 5.

Gene function	Reference
ADG2 catalyzes rate limiting step in starch biosynthesis	1
AGL18 and AGL15 act in a redundant fashion as repressors of the floral transition	2
AGL19 activates LFY in the FLC-independent vernalization pathway	3
AHL18 acts redundantly with AHL22, AHL27 and AHL29 in the regulation of flowering	4
ARF8 acts redundantly with ARF6, AS1 and AS2 to control stamen elongation and flower maturation	5
BOP1 and BOP2 promotes floral meristem fate in a pathway targetting AP1 and AGL24	6
BRI1 represses FLC expression through brassinosteroid signal transduction pathway	7-9
CAL acts as a floral homeotic gene encoding a MADS domain protein homologous to AP1	10
CO is regulated by the circadian clock genes, and directly binds to and activates FT in LD	11-13
COL2 acts as a homologous to the flowering-time gene CO	14
COP1 acts as an E3 Ubiquitin ligase ubiquitinating CO protein	15,16
CRY2 represses *COP1* expression and promotes CO	17
CUL3A interacts with other components of E3 ligase complex functioning in RUB-modification	18
EBS participates in the regulation of flowering time by specifically repressing the expression of FT	19
EIP9 interacts with EMF1, EIP1 or EIP6 to regulate flowering time	20
ELF5 affects flowering time independent of FLC	21
FKF1 binds to GI and represses CDF1	22,23
FLC suppresses FT and SOC1	24,25
FLX4 positively regulates FLC expression	26,27
FRY1 probably positively regulates FT transcript	28
FUL acts as a modulator of the activity of the floral regulators SVP and SOC1	29,30
FYPP3 functions primarily through the Long-Day flowering pathway	31
GI positively regulates CO together with FKF1	32
GID1A, GID1B and GID1C acts as GA receptors	33-36
GRP7 and GRP8 promote early flowering	37,38
HDA5 represses FLC expression through histone deacetylation	39
HYL1 acts as a nuclear dsRNA binding protein involved in mRNA cleavage	40,41
IBM1 positively flowering through H3K9 demethylation	42,43
KNU maintains balance e of proliferation and differentiation in flower development	44
LD negatively represses FLC expression	45
LDL2 represses FLC expression through H3K4 methylation	46
LFY and AP1 act as a floral meristem identy gene	47-50
MAF2 represses SOC1 expression	51,52
MBD9 activates FLC through acetylation of FLC chromatin	53
AT1G65010, microtubule-associated protein repressing flowering	54
MSI1 promotes CO expresssion	55,56
NAC050 associates with the histone H3K4 demethylase JMJ14 and represses LFY	57
NUA negatively regulates flowering	58
PGM1 promotes flowering in carbohydrate metabolism pathway	59
PI4Kγ3 encodes a type II phosphoinositide 4-kinase and activates FLC	60
PNY promotes LFY expression	61
PUCHI promotes LFY expression	62

RLT1 interacts with ISWI and represses SOC1 expression	63
SDG27 dynamically regulates FLOWERING LOCUS C activation via H3K4 trimethylation	64
SOB7 negatively regulates FLC expression	65
SOC1 integrates signals from multiple flowering pathways and promotes LFY expression	66-68
SPA binds to COP1 and regulates CO protein levels	69-71
SPL activates SOC1, FUL and LFY	72-74
SQN regulates redundantly with REBELOTE, SQUINT, and ULTRAPETALA1 flowering	75
SVP interacts with FLC for the repression of SOC1in the apex	76
TIR1 activates LFY through auxin signaling transduction	77
TOE3 negatively regulates LFY	78
TPS1 positively regulates flowering through carbohydrate metabolism pathway	79
UBC1 activates FLC expression through H2B monoubiquitination	80
UGT87A2 promotes flowering through repressing FLC	81
UPF1 represses flowering through nonsense-mediated mRNA decay	82,83
VGT1 positively regulates flowering through carbohydrate metabolism pathway	84
VIN3 shuts down FLC transcript through histone methylation	85
VOZ1/2 negatively regulates FLC expression	86
VRN1 maintains the methylated state of FLC chromatin	87

Supplementary Table S4. Functional description of genes involved in petal development in this study.

Gene function	Reference
ADG2 redundantly promotes petal development with ANT	1
ARF2 represses petal development through repressing cell devision	2
ARF3 negatively petal development	3
ARP6 as a component of SWR1 complex restricts excessive development of petals	4
AS1/2 and JAGGED are required for sepal and petal organogenesis	5
ASK1/2/3 and UFO are required for normal function of LFY protein	6-8
AXL1 redundantly modifies CUL1 with AXR1	9,10
CHR11 associates with AP1, AP3, PI and SEP3	11,12
CUC1/2 and EEP1 involve auxin influ required for petal development	13
EMF1 forms PRC1 with LHP1, and represses AP3 and AG expression	14,15
FIL is necessary for the accurate expression of AG, AP3 and PI	16
IFL is required for the precise number of petals	17
JMJ12/REF6 interacts with AP1	18
KNATM ensures the normal development of petal	19,20
MED8 regulates cell size of petal	21
MSI1 forms PRC2 with FIE, SWN and EMF2, and represses AG、PI and AP3 expression	22
PLP is involved in the negative regulation of petal onset through protein farnesylation	23
PNY acts as a positive regulator of petal development	24
PTL regulates initiation and orientation of petals	25
RBE acts downstream of AP1 and PTL genes, and is required for early development of petals	26
SEF as a component of SWR1 complex ensures proper petal development	4
SEP1/2/3 as MADS-box genes is required to specify petals	27
TOP1ALPHA interacts with LFY, AP1 and AP2, and ensures the normal number of petals	28
TPL forms a complex with AP2 and HDA19, and represses AG, AP3 and PI expression	29
TSL ensures the normal number of petals	30
WIG involve in protein farnesylation restricts the number of petals	31

Supplementary Table S5. List of DEGs underlying the CIs of *qPD.A9-2*, *qPD.C8-2* and *qPD.C8-3*.

QTL	Gene id	Fold Change	
		RNA-seq	qRT-PCR
qPD.A9-2	BnaA09g36340D	1.908977369	0.701
qPD.A9-2	**BnaA09g36370D**	**0.270687468**	**0.161**
qPD.A9-2	BnaA09g36390D	0	1.022
qPD.A9-2	BnaA09g36450D	2.58972696	0.511
qPD.A9-2	BnaA09g36500D	0.431161421	1.823
qPD.A9-2	**BnaA09g36510D**	**2.306811309**	**2.936**
qPD.A9-2	**BnaA09g36520D**	**2.154420705**	**5.815**
qPD.A9-2	BnaA09g36540D	2.289924448	0.844
qPD.A9-2	BnaA09g36610D	3.08656035	0.508
qPD.A9-2	BnaA09g36620D	3.072472292	0.568
qPD.A9-2	**BnaA09g36660D**	**1.910685064**	**1.277**
qPD.A9-2	**BnaA09g36670D**	**1.760786513**	**1.272**
qPD.A9-2	**BnaA09g36680D**	**3.446901327**	**24.658**
qPD.A9-2	**BnaA09g36710D**	**3.113419548**	**1.718**
qPD.A9-2	**BnaA09g36720D**	**2.43986276**	**3.196**
qPD.A9-2	BnaA09g36740D	0.085495956	0.978
qPD.A9-2	BnaA09g36760D	10.46282114	1.065
qPD.A9-2	**BnaA09g36770D**	**2.131250245**	**1.294**
qPD.A9-2	**BnaA09g36810D**	**2.676226088**	**3.083**
qPD.A9-2	BnaA09g36870D	4.123301625	0.154
qPD.A9-2	BnaA09g36900D	86.26479471	0.492
qPD.A9-2	**BnaA09g37060D**	**0.546953466**	**0.456**
qPD.A9-2	BnaA09g37090D	0.328303037	1.302
qPD.A9-2	BnaA09g37140D	4.271965512	0.861
qPD.A9-2	**BnaA09g37200D**	**4.478451555**	**1.704841906**
qPD.A9-2	**BnaA09g37250D**	**18.1651327**	**6.010044322**
qPD.A9-2	Novel01045	3.190317482	0.747
qPD.A9-2	**BnaA09g37270D**	**0.458851745**	**0.463**
qPD.A9-2	BnaA09g37290D	2.028762523	1.073
qPD.A9-2	***BnaA09g37300D***	**2.484232152**	**1.338482584**
qPD.A9-2	**BnaA09g37350D**	**4.820223361**	**3.052**
qPD.A9-2	BnaA09g37400D	2.920671295	0.944
qPD.A9-2	**BnaA09g37520D**	**0**	**0.107**
qPD.C8-2	Novel02823	0.360682167	0.851
qPD.C8-2	BnaC08g10190D	+∞	1.038
qPD.C8-2	BnaC08g10310D	0.092046074	2.161
qPD.C8-2	Novel02888	0.018569773	2.739
qPD.C8-2	BnaC08g10840D	0.002283876	22.812
qPD.C8-2	BnaC08g10880D	0.480930391	2.729
qPD.C8-2	BnaC08g10890D	0.324907286	1.701

qPD.C8-2	***BnaCnng35110D***	**1.72573387**	**2.504**
qPD.C8-2	BnaC08g10960D	0.487982055	1.738
qPD.C8-2	BnaC08g11040D	0.219318589	5.699
qPD.C8-2	**BnaC08g11110D**	**1.831924441**	**3.353**
qPD.C8-2	**BnaC08g11240D**	**9.807599046**	**3.429**
qPD.C8-2	**BnaC08g11580D**	**0**	**0.271**
qPD.C8-2	**BnaC08g11590D**	**0**	**0.484**
qPD.C8-2	BnaC08g11600D	0.013307653	1.741
qPD.C8-2	**BnaC08g11790D**	**0.440526282**	**0.485**
qPD.C8-2	BnaC08g11890D	3.326949575	1.038
qPD.C8-2	BnaC08g12000D	6.124109166	0.892
qPD.C8-3	**Novel02901**	**2.145181921**	**2.628**
qPD.C8-3	**BnaC08g13750D**	**9.497843602**	**1.892**
qPD.C8-3	BnaC08g13780D	0.558368709	0.896
qPD.C8-3	BnaC08g13920D	0.464934697	1.159
qPD.C8-3	BnaC08g13940D	0.216029464	0.846388003
qPD.C8-3	BnaC08g13960D	0.32585454	1.546
qPD.C8-3	***BnaC08g13970D***	**0.045898792**	**0.442**
qPD.C8-3	***BnaC08g14080D***	**3.85946266**	**1.28**
qPD.C8-3	***BnaC08g14110D***	**7.127025074**	**1.94134949**
qPD.C8-3	***BnaC08g14130D***	**11.60037871**	**1.603431283**
qPD.C8-3	**BnaC08g14290D**	+∞	**7.168**
qPD.C8-3	**Novel02902**	**841.7219911**	**4.104**
qPD.C8-3	**BnaC08g14360D**	+∞	**1.401**
qPD.C8-3	BnaC08g14420D	2.472721912	0.933
qPD.C8-3	**BnaC08g14450D**	**4.184919872**	**1.460651278**
qPD.C8-3	**BnaC08g14670D**	**2.23969146**	**2.801420212**

The expression patterns of 67 DEGs identified in the RNA-seq assay and located in the CIs of *qPD.A9-2*, *qPD.C8-2* and *qPD.C8-3* were verified in young inflorescences collected from APL01 and Holly at the same developmental stages as used for the RNA-seq assay by qRT-PCR.

The bold genes have the same expression changes between lines APL01 and PL01 as between APL01 and Holly.

Genes indicated in italics were the potential candidate genes regulating the petal development of line APL01.

"+∞" indicates that gene is only expressed in line APL01.

Supplementary Table S6. Gene-specific primers used for qRT-PCR in this study.

Primer name	Forward primer (5'to 3')	Reverse primer (5'to 3')
ACTIN	CGAGGCTCCTCTTAACCCAAAGG	CACCAGAATCCAGCACAATACCG
AIL5	ATAGATGGACGGGAAGGTATG	AAATTGGTGGTAGCAGCAGTA
ARF2	GCGGAGATAACTCAGTCTAACC	CATAAGCACCACAAACGAAAG
ARF3	TCAGGCTGGCAGCTTGTA	TGGGTGAGGTTCTGTGGG
ARP6	TCACTCCTCCGCATCACC	AGAAGCCACAATCCACGAC
AS1	GAAGACAAGTAGACAAAGGGAGA	ATCATAGTAGGTGTTTGGTGGTT
AS2	CCATCATTATCATCACCACCAG	TCAACAGTACGGCGACCA
ASK1	GTCTTTCGAGGTCGATGAGG	TTAGCCGCCAGGATGAGT
ASK2	TCCTGGCTGCTAACTATCTGA	AAAGCCCATTGGTTCTCC
ASK3	AGAACGACTACACGCCTGAG	AAAGCACAAAGACGCAAACT
AXL1	GTAGCTGAGAAGCGAACCAC	GATTCCACCAAGAACGAGATT
AXR1	TGAAGAAGGAAGAGGCAACA	ACTCCACCGAGGACGAGA
CHR11	TTTGAACTGGCTCATCCG	AACTAGCAGGTCATCACGAAT
CO	GTTCACGCTGCCAATCTTC	CTGTCATCGCTGTTGTCTCAC
COL2	AAACAGGGTAACTGGTAAGTGAT	TACACGAATAAAGTAACGAGGG
COP1	ACTTGCCAGTTCGGACATT	GCATCGTGGGACTATCGC
CRY2-1	GACCAGCAAGTACCATCGG	CACCAGGGAGCAAGAATGA
CRY2-2	CGGGGAAATAAGCGTCAGA	ATCCATCCAGTAGCCCAAAG
CUC1	TCTTCTTCTACCGCTTCCG	ATAAACTGACCAAACGCCAC
EMF1	ATGGTGGCGTTGTATGAGC	TTTGCTTTGACTAGGGATAACC
FKF1-1	CTATACCAAGATCATGGCATAGC	AGCACGGCTCATTCTCACTA
FKF1-2	AAAGCGAGATCCCTCCTTC	ACTCATTAAACCGCCGACA
FT-1	CCAAGTCCAAGCAACCCTC	TTCTCAGCGAACGCACGA
FT-2	TGATATCCCAGCGACAACTG	TGTATAGGGAAGCAAACTCACG
FT-3	AAGCCCAAGCAACCCGTA	TTCCTCCGCAGCCATTCT
GI	TGCTGAGTTGGTCCGTAATC	TGCTGCTCCGTCTTGTAAAT
MSI1	GAGTTTGGTGGGTTTGGC	GCTTAGACGGATGCTTGCT
PFT1	TTTCGTCCATACAATTCGC	TGATAAACGGCTTTCAGGTAG
PHYB	TCCCTTAGGCGTTGCTCC	CTTTGCCCATCATCTTTATCC
PRR5-1	AAGCCCGAGTTCCGTTAG	CGTTGCTCAGCCAGTTTC
PRR5-2	AGAAACGGACAAGACAACAGA	AAGGGAGATACCGAGCATT
SPA1	TGGTTGACTCAGCCCATTC	CCAGTTGAAGGAGCAGATGA
SPA3-1	CTTTCTGCTTGTGGCTACTGC	AGAAGTGAAACTGTATCCCTCAATC
SPA3-2	GATATTTCATCAGCAGACCCTAC	TGGGGTTCCTAAGATCGTAGT
BnaA09g36340D	TCCCTGGAAGTGGACTAATG	ATCTGTGCCAATCAAATGAG
BnaA09g36370D	CGGCGAATAGGGAGAAGT	GCAGGACCAACAATCATAAGG
BnaA09g36390D	TACTCCGCTTTACCAGACG	AAGAGCAGCCATTCCACTA
BnaA09g36450D	TCACGAGCAGTGTAGTAGGG	GTAATTTGTAGTTGGTTGGGTC
BnaA09g36500D	CAAGAAGCGATGAGCAGTAG	ATTTGCGGAAGAAGAGGC
BnaA09g36510D	ACATGCCTGCATCCCACT	GCCATTGCTTGTTCTTCCTA
BnaA09g36520D	GTGGTTACTCGCCGTGCT	GATCTGACAACTGCGTATTCTGA
BnaA09g36540D	TATGACTCTGAAGGAGCGTGTA	CGAATTCAAACTCTATTGTGGC

BnaA09g36610D	AGATTCACGGATGAGTACAAGA	TCCAATGCTGCGTAGACC
BnaA09g36620D	GTTCCGTCCCGAGTCTTT	CATGTTCCTCTTTACCCGTAG
BnaA09g36660D	ATCCTGGGATCACCTACTGA	GTACACTGCCACCAGAATGA
BnaA09g36670D	TCTCAGCCTTCTTTACGCC	CTTATCGGGATGGACTTTCA
BnaA09g36680D	AGTCCTCAAGTATCTTCAAACACTG	CCACGAGTCAAATCAATCCC
BnaA09g36710D	GGTAGAAAGATCCCGGTTAA	AAGGAAGTGTCGGAATGG
BnaA09g36720D	GAAGGGATTTGAAGTGGTGG	AGAATCTAACTGAGAAGCCGAAC
BnaA09g36740D	TGGAAGGGCTGGTAGAGTT	CCAAAGACCTGCTGATGAATA
BnaA09g36760D	CTATTACCATCCTCCTTCCG	CTCTGGCGTCTTCATACACTT
BnaA09g36770D	TTCCTGGTCAGCCGTGTAAT	CAACCTAAGTGTTTTAACGCATG
BnaA09g36810D	TGTCTTGGAGGGTCTTCGG	TGGGAGCATCATCTCCGTT
BnaA09g36870D	ATTCTGCTGACCGATGTGG	CTGTCTCCTCCCATCAATAATACT
BnaA09g36900D	AAAGCCATGATACCTCCAT	AACTTAATCGCACTGAAACC
BnaA09g37060D	GTCCCAGGCTGTGATAAGAT	CTGAGGCTAATAAGGTGTCG
BnaA09g37090D	TCCAGTTCGCAGATTACCG	AAGCACCACCTCTTCCACG
BnaA09g37140D	TCCATTTCCATTGCCAGAC	TATTTCCACTTGCATAGAGCC
BnaA09g37200D	GGACGAGTTTGCCTGTGAG	CAACACCACCATTATCCCC
BnaA09g37250D	GGAAGTCAACTCTACGGAAACA	GTCGAAAGGGTCTTCAGGAT
Novel01045	CTCCCTCAGTGTTACCTTGTGG	ACGACTCGCCCATTGCTC
BnaA09g37270D	GCGTTACTTCATTGCAGACG	CGGAGATTGATCGGAGACA
BnaA09g37290D	TTCCATTTACCGAACCATC	ACCATAGCAAGTCGCTCC
BnaA09g37300D	TGGCATTGAAGAGGGAGG	CCTGAGAGAATCCCCCTTG
BnaA09g37350D	CACTTGCCAAACTTCATTCC	AGAGGTTTCGTTTCCCACT
BnaA09g37400D	GGAAGATGAGGCCGAACA	CGATAGAATTGCAGAGGGTAAG
BnaA09g37520D	ACAGCCGCAGACCACATAC	GAGACCATTCGTTACCCAAG
Novel02823	ACAACGCCACTTGGAAAC	TAAAGCAAACCCGAGCAG
BnaC08g10190D	CTGTGACGGGACACGAGAAT	CCACCAAGAACCCAACCAA
BnaC08g10310D	ACTCGCTCGTCTTCGTTC	GACCACAGCCAAGCATTT
Novel02888	TCTCCTCCTCGTCTCACCA	TAGCATTGCCCCTCTTCG
BnaC08g10840D	CTGCTCCCATTGGAGATT	TTTGCCAGTAGTGCTAGATTG
BnaC08g10880D	TCGCTCGCTTGCTATCCC	TCCCATCAACCCAATCCC
BnaC08g10890D	AGGTTCGGACTCTGGTTCTC	TAAACATTGGAGATGAAAGAGTAGA
BnaCnng35110D	CAAAGCAAGCCAAGTCTCC	GAAAGCAGCGGTATTAGCATC
BnaC08g10960D	AAATCAGGCAAAGAAGGAGG	AGGTTATTACCCGACCAAGC
BnaC08g11040D	GATTACTGACAGATTTATGGGAGG	CGATGAATGTAGCGAGGAAGA
BnaC08g11110D	TTGCGGAGGGTGTTATCG	TCCTCCGTTTCCTTTACCG
BnaC08g11240D	GATTACAACTTACGAGGGTCAACAC	TCATATTCACTTCCTTGGCGAT
BnaC08g11580D	CGCTTCCCGATTCGCTAT	GTCCGTGTCAGGCTGATGAG
BnaC08g11590D	TTTTGACAGCTTAGTTGGGATG	CAAACACACTCAGTTGGAAATCTC
BnaC08g11600D	CGAAAACTTTCTCAGCCACAT	AGCAAGACAATCGCAATCCT
BnaC08g11790D	GTGCTTCGGACGGTGATT	TCTGCTTTCTACCTGGTGAGTG
BnaC08g11890D	CCTTAATGCACGAAATCGC	CTCCAAATCACTTGCCTCAC
BnaC08g12000D	TGAGCCCGACCAGGTTATT	AGTTCACTACCCCACGATGC
Novel02901	CAGTGCCGAGTTACCAGC	GACCGTTGCATCATCAGTTC

BnaC08g13750D	TGGATTGGGTTGTGACTGG	CAACGGCTTCACTGCTACTT
BnaC08g13780D	GTTCTCCATTTCGTGCTCC	CGCCCTCTAATAAACACCCTA
BnaC08g13920D	GAAGTCTCAGTGCCGTTGGA	GTGGGATACTGCCTTGAATCTG
BnaC08g13940D	CTTCGTCTCGCCGTCTTC	TTTGGAGTGCGGTTGGTC
BnaC08g13960D	TGGCTCTTCGCTGCTTACT	ACATTAGAACCCTACCGTCTTC
BnaC08g13970D	CAAGGAAGGTATTGCTGCTC	AGAATAGTCCCATTGGTTGC
BnaC08g14080D	TGACTGCGTAGAAGGTGGTT	GTCTCCCTGCTATGACAAACA
BnaC08g14110D	GGAGATAATGCCGCTAAGG	CAAGATCACCCGAGGAGAC
BnaC08g14130D	AAGGTAACGATGAGGAGGCT	TTCACAGACGAAATATCAGGAA
BnaC08g14290D	CATCAGCGGTGGTAATCG	TCTCCTTCTTGTGAGCGTTT
Novel02902	GAACAAACGTGGTGGGAG	AAACGCAACGGAGACTGG
BnaC08g14360D	CTTTGCCTGCAAGGAGTG	GGATGTGGGCTCTGATTGT
BnaC08g14420D	GTGAAAGTCATCAGCACGAA	ATCAACAGCAAGCCTCATAG
BnaC08g14450D	TGAGGAGACGGGAGGAGT	GTTGGGATTGGCAGGAAC
BnaC08g14670D	AGCCTCAGATCCCTCCCT	GTCGTCTTCGCACTCCACT

SUPPLEMENTARY REFERENCES

Selected references for the gene functions summarized in Supplementary Table S3

1. Wang, S. M. *et al.* adg2-1 represents a missense mutation in the ADPG py-rophosphorylase large subunit gene of *Arabidopsis thaliana*. *Plant J* **11**, 1121 – 1126 (1997).
2. Adamczyk, B. J., Lehti-Shiu, M. D. & Fernandez, D. E. The MADS domain factors *AGL15* and *AGL18* act redundantly as repressors of the floral transition in *Arabidopsis. Plant J* **50**, 1007-1019 (2007).
3. Schönrock, N. *et al.* Polycomb-group proteins repress the floral activator *AGL19* in the FLC-independent vernalization pathway. *Genes Dev* **20**, 1667-1678 (2006).
4. Yun, J., Kim, Y. S., Jung, J. H., Seo, P. J. & Park, C. M. The AT-hook motif-containing protein AHL22 regulates flowering initiation by modifying *FLOWERING LOCUS T* chromatin in *Arabidopsis*. *J Biol Chem* **287**, 15307-15316 (2012).
5. Tabata, R. *et al. Arabidopsis AUXIN RESPONSE FACTOR 6* and 8 Regulate Jasmonic Acid Biosynthesis and Floral Organ Development via Repression of Class 1 *KNOX* Genes. *Plant Cell Physiol* **51**, 164-175 (2009).
6. Xu, M. *et al. Arabidopsis BLADE-ON-PETIOLE1* and 2 promote floral meristem fate and determinacy in a previously undefined pathway targeting *APETALA1* and *AGAMOUS-LIKE24*. *Plant J* **63**, 974-989 (2010).
7. Li, J. & Chory, J. A putative leucine-rich repeat receptor kinase involved in brassinosteroid signal transduction. *Cell* **90**, 929-938 (1997).
8. Noguchi, T. *et al.* Brassinosteroid-insensitive dwarf mutants of *Arabidopsis* accumulate brassinosteroids. *Plant Physiol* **121**, 743-752 (1999).
9. Domagalska, M. A. *et al.* Attenuation of brassinosteroid signaling enhances *FLC* expression and delays flowering. *Development* **134**, 2841-2850 (2007).
10. Purugganan, M. D. & Suddith, J. I. Molecular population genetics of the *Arabidopsis CAULIFLOWER* regulatory gene: nonneutral evolution and naturally occurring variation in floral homeotic function. *Proc Natl Acad Sci* **95**, 8130-8134 (1998).
11. Rédei, G. P. Supervital mutants of *Arabidopsis*. *Genetics* **47**, 443 – 460 (1962).
12. Putterill, J., Robson, F., Lee, K., Simon, R. & Coupland, G. The *CONSTANS* gene of *Arabidopsis* promotes flowering and encodes a protein showing similarities to zinc finger transcription factors. *Cell* **80**, 847 – 857 (1995).
13. Suárez-López, P. *et al. CONSTANS* mediates between the circadian clock and the control of flowering in *Arabidopsis*. *Nature* **410**, 1116 – 1120 (2001).
14. Chia, T. Y., Müller, A., Jung, C. & Mutasa-Göttgens, E. S. Sugar beet contains a large *CONSTANS-LIKE* gene family including a *CO* homologue that is independent of the early-bolting (B) gene locus. *J Exp Bot* **59**, 2735-2748 (2008).
15. Jang, S. *et al. Arabidopsis COP1* shapes the temporal pattern of *CO* accumulation conferring a photoperiodic flowering response. *EMBO J* **27**, 1277 – 1288 (2008).
16. Liu, L. J. *et al.* COP1-mediated ubiquitination of *CONSTANS* is implicated in cryptochrome regulation of flowering in *Arabidopsis*. *Plant Cell* **20**, 292-306 (2008).
17. Tóth, R. *et al.* Circadian clock-regulated expression of phytochrome and cryptochrome genes in *Arabidopsis*. *Plant Physiol* **127**, 1607 – 1616 (2001).
18. Dieterle, M. *et al.* Molecular and functional characterization of *Arabidopsis* Cullin 3A. *Plant J* **41**, 386-399 (2005).

19. Piñeiro, M., Gómez-Mena, C., Schaffer, R., Martínez-Zapater, J. M. & Coupland, G. *EARLY BOLTING IN SHORT DAYS* is related to chromatin remodeling factors and regulates flowering in *Arabidopsis* by repressing *FT*. *Plant Cell* **15**, 1552-1562 (2003).
20. Park, H. Y. *et al*. *EMF1* interacts with *EIP1*, *EIP6* or *EIP9* involved in the regulation of flowering time in *Arabidopsis*. *Plant Cell Physiol* **52**, 1376-1388 (2011).
21. Noh, Y. S., Bizzell, C. M., Noh, B., Schomburg, F. M. & Amasino, R. M. *EARLY FLOWERING 5* acts as a floral repressor in *Arabidopsis*. *Plant J* **38**, 664-672 (2004).
22. Sawa, M., Nusinow, D. A., Kay, S. A. & Imaizumi, T. *FKF1* and *GIGANTEA* complex formation is required for day-length measurement in *Arabidopsis*. *Science* **318**, 261 – 265 (2007).
23. Nelson, D. C., Lasswell, J., Rogg, L. E., Cohen, M. A. & Bartel, B. *FKF1*, a clock-controlled gene that regulates the transition to flowering in *Arabidopsis*. *Cell* **101**, 331 – 340 (2000).
24. Michaels, S. D. & Amasino, R. M. *FLOWERING LOCUS C* encodes a novel MADS domain protein that acts as a repressor of flowering. *Plant Cell* **11**, 949 – 956 (1999).
25. Helliwell, C. A., Wood, C. C., Robertson, M., James Peacock, W. & Dennis, E. S. The *Arabidopsis* FLC protein interacts directly in vivo with *SOC1* and *FT* chromatin and is part of a high-molecularweight protein complex. *Plant J* **46**, 183 – 192 (2006).
26. Lee, J. & Amasino, R. M. Two *FLX* family members are non-redundantly required to establish the vernalization requirement in *Arabidopsis*. *Nat Commun* **4**, 2186 (2013).
27. Ding, L., Kim, S. Y. & Michaels, S. D. *FLOWERING LOCUS C EXPRESSOR* family proteins regulate *FLOWERING LOCUS C* expression in both winter-annual and rapid-cycling *Arabidopsis*. *Plant Physiol* **163**, 243-252 (2013).
28. Kim, B. H. & von Arnim, A. G. *FIERY1* regulates light-mediated repression of cell elongation and flowering time via its 3'(2'),5'-bisphosphate nucleotidase activity. *Plant J* **58**, 208-219 (2009).
29. Torti. S. & Fornara, F. *AGL24* acts in concert with *SOC1* and *FUL* during *Arabidopsis* floral transition. *Plant Signal Behav* **7**, 1251-1254 (2012).
30. Balanzà, V., Martínez-Fernández, I. & Ferrándiz, C. Sequential action of *FRUITFULL* as a modulator of the activity of the floral regulators *SVP* and *SOC1*. *J Exp Bot* **65**, 1193-1203 (2014).
31. Kim, D. H. *et al*. A phytochrome-associated protein phosphatase 2A modulates light signals in flowering time control in *Arabidopsis*. *Plant Cell* **14**, 3043-3056 (2002).
32. Rubio, V. & Deng, X. W. Standing on the shoulders of *GIGANTEA*. *Science* **318**, 206 – 207 (2007).
33. Ueguchi-Tanaka, M. *et al*. *GIBBERELLIN INSENSITIVE DWARF1* encodes a soluble receptor for gibberellin. *Nature* **437**, 693 – 698 (2005).
34. Griffiths, J. *et al*. Genetic characterization and functional analysis of the GID1 gibberellin receptors in *Arabidopsis*. *Plant Cell* **18**, 3399 – 3414 (2006).
35. Willige, B. C. *et al*. The DELLA domain of *GA INSENSITIVE* mediates the interaction with the *GA INSENSITIVE DWARF1A* gibberellin receptor of *Arabidopsis*. *Plant Cell* **19**, 1209 – 1220 (2007).
36. Murase, K., Hirano, Y., Sun, T. P. & Hakoshima, T. Gibberellininduced DELLA recognition by the gibberellin receptor GID1. *Nature* **456**, 459 – 463 (2008).
37. Heintzen, C., Nater, M., Apel, K. & Staiger, D. A*tGRP7*, a nuclear RNA-binding protein as a component of a circadian-regulated negative feedback loop in *Arabidopsis thaliana*. *Proc Natl Acad Sci* **94**, 8515-8520 (1997).
38. Schmal, C., Reimann, P. & Staiger, D. A circadian clock-regulated toggle switch explains

AtGRP7 and *AtGRP8* oscillations in *Arabidopsis thaliana*. *PLoS Comput Biol* **9**, e1002986 (2013).
39. Luo, M. *et al*. Regulation of flowering time by the histone deacetylase HDA5 in *Arabidopsis*. *Plant J* **82**, 925-936 (2015).
40. Lu, C. & Fedoroff, N. A mutation in the *Arabidopsis HYL1* gene encoding a dsRNA binding protein affects responses to abscisic acid, auxin, and cytokinin. *Plant Cell* **12**, 2351-2366 (2000).
41. Vazquez, F., Gasciolli, V., Crété, P. & Vaucheret, H. The nuclear dsRNA binding protein HYL1 is required for microRNA accumulation and plant development, but not posttranscriptional transgene silencing. *Curr Biol* **14**, 346-351 (2004).
42. Fan, D. *et al*. *IBM1*, a JmjC domain-containing histone demethylase, is involved in the regulation of RNA-directed DNA methylation through the epigenetic control of *RDR2* and *DCL3* expression in *Arabidopsis*. *Nucleic Acids Res* **40**, 8905-8916 (2012).
43. Lei, M. *et al*. *Arabidopsis EDM2* promotes *IBM1* distal polyadenylation and regulates genome DNA methylation patterns. *Proc Natl Acad Sci* **111**, 527-532 (2014).
44. Sun, B., Xu, Y., Ng, K. H. & Ito, T. A timing mechanism for stem cell maintenance and differentiation in the *Arabidopsis* floral meristem. *Genes Dev* **1791-1804**(2009).
45. Lee, I. *et al*. Isolation of *LUMINIDEPENDENS*: a gene involved in the control of flowering time in *Arabidopsis*. *Plant Cell* **6**, 75 – 83 (1994).
46. Jiang, D., Yang, W., He, Y. & Amasino, R. M. *Arabidopsis* relatives of the human lysine-specific Demethylase1 repress the expression of *FWA* and *FLOWERING LOCUS C* and thus promote the floral transition. *Plant Cell* **19**, 2975-2987 (2007).
47. Weigel, D., Alvarez, J., Smyth, D. R., Yanofsky, M. F. & Meyerowitz, E. M. *LEAFY* controls floral meristem identity in *Arabidopsis*. *Cell* **69**, 843 – 859 (1992).
48. Irish, V. F. & Sussex, I. M. Function of the *APETALA1* gene during *Arabidopsis* floral development. *Plant Cell* **8**, 741 – 753 (1990).
49. Bowman, J., Alvarez, J., Weigel, D., Meyerowitz, E. M. & Smyth, D. R. Control of flower development in *Arabidopsis* thaliana by *APETALA1* and interacting genes. *Development* **119**, 721 (1993).
50. Wagner, D., Sablowski, R. W. & Meyerowitz, E. M. Transcriptional activation of *APETALA1* by *LEAFY*. *Science* **285**, 582-584 (1999).
51. Rosloski, S. M. *et al*. Functional analysis of splice variant expression of *MADS AFFECTING FLOWERING 2* of *Arabidopsis thaliana*. *Plant Mol Biol* **81**, 57-69 (2013).
52. Ratcliffe, O. J., Kumimoto, R. W., Wong, B. J. & Riechmann, J. L. Analysis of the *Arabidopsis MADS AFFECTING FLOWERING* gene family: *MAF2* prevents vernalization by short periods of cold. *Plant Cell* **15**, 1159-1169 (2003).
53. Peng, M., Cui, Y., Bi, Y. M. & Rothstein, S. J. *AtMBD9*: a protein with a methyl-CpG-binding domain regulates flowering time and shoot branching in *Arabidopsis*. *Plant J* **46**, 282-296 (2006).
54. Alonso, J. M. *et al*. Genome-wide insertional mutagenesis of *Arabidopsis thaliana*. *Science* **301**, 653-657 (2003).
55. Hennig, L., Taranto, P., Walser, M., Schönrock, N. & Gruissem, W. *Arabidopsis MSI1* is required for epigenetic maintenance of reproductive development. *Development* **130**, 2555-2565 (2003).
56. Steinbach, Y. & Hennig, L. *Arabidopsis MSI1* functions in photoperiodic flowering time control. *Front Plant Sci* **5**, 77 (2014).
57. Ning, Y. Q. *et al*. Two novel NAC transcription factors regulate gene expression and flowering time by associating with the histone demethylase JMJ14. *Nucleic Acids Res* **43**, 1469-1484 (2015).

58. Xu, X. M. *et al*. *NUCLEAR PORE ANCHOR*, the *Arabidopsis* homolog of Tpr/Mlp1/Mlp2/megator, is involved in mRNA export and SUMO homeostasis and affects diverse aspects of plant development. *Plant Cell* **19**, 1537-1548 (2007).
59. Caspar, T., Huber, S.C. & Somerville, C. Alterations in growth, photosynthesis, and respiration in a starchless mutant of *Arabidopsis thaliana L.* deficient in a chloroplast phosphoglucomutase activity. *Plant Physiol* **79**, 11 – 17 (1985).
60. Akhter, S. *et al*. Role of *Arabidopsis AtPI4K γ3*, a type II phosphoinositide 4-kinase, in abiotic stress responses and floral transition. *Plant Biotechnol J* **14**, 215-230 (2016).
61. Kanrar, S., Bhattacharya, M., Arthur, B., Courtier, J. & Smith, H. M. Regulatory networks that function to specify flower meristems require the function of homeobox genes *PENNYWISE* and *POUND-FOOLISH* in *Arabidopsis*. *Plant J* **54**, 924-937 (2008).
62. Karim, M. R., Hirota, A., Kwiatkowska, D., Tasaka, M. & Aida, M. A role for *Arabidopsis PUCHI* in floral meristem identity and bract suppression. *Plant Cell* **21**, 1360-1372 (2009).
63. Li, G., Zhang, J., Li, J., Yang, Z., Huang, H. & Xu, L. ISWI chromatin remodeling factors and their interacting RINGLET proteins act together in controlling the plant vegetative phase in *Arabidopsis*. *Plant J* **72**, 261-270 (2012).
64. Pien, S. *et al*. *ARABIDOPSIS TRITHORAX1* dynamically regulates *FLOWERING LOCUS C* activation via histone 3 lysine 4 trimethylation. *Plant Cell* **20**, 580-588 (2008).
65. Turk, E. M. *et al*. *BAS1* and *SOB7* act redundantly to modulate *Arabidopsis* photomorphogenesis via unique brassinosteroid inactivation mechanisms. *Plant J* **42**, 23-34 (2005).
66. Moon, J. *et al*. The *SOC1* MADS-box gene integrates vernalization and gibberellin signals for flowering in *Arabidopsis*. *Plant J* **613 – 623**(2003).
67. Lee, J., Oh, M., Park. H. & Lee, I. *SOC1* translocated to the nucleus by interaction with *AGL24* directly regulates leafy. *Plant J* **55**, 832 – 843 (2008).
68. Lee, J. & Lee, I. Regulation and function of *SOC1*, a flowering pathway integrator. *J Exp Bot* **61**, 2247 – 2254 (2010).
69. Hoecker, U. & Quail, P. H. The phytochrome A-specific signaling intermediate *SPA1* interacts directly with *COP1*, a constitutive repressor of light signaling in *Arabidopsis*. *J Biol Chem* **276**, 38173 – 38178 (2001).
70. Laubinger, S. & Hoecker, U. The SPA1-like proteins *SPA3* and *SPA4* repress photomorphogenesis in the light. *Plant J* **35**, 373 – 385 (2003).
71. Laubinger, S. *et al*. *Arabidopsis* SPA proteins regulate photoperiodic flowering and interact with the floral inducer *CONSTANS* to regulate its stability. *Development* **133**, 3213-3222 (2006).
72. Yamaguchi, A. *et al*. The microRNA regulated SBP-Box transcription factor *SPL3* is a direct upstream activator of *LEAFY*, *FRUITFULL*, and *APETALA1*. *Dev Cell* **17**, 268-278 (2009).
73. Jung, J. H., Ju, Y., Seo, P. J, Lee, J. H. & Park, C. M. The SOC1-SPL module integrates photoperiod and gibberellic acid signals to control flowering time in *Arabidopsis*. *Plant J* **69**, 577-588 (2012).
74. Jorgensen, S. A. & Preston, J. C. Differential *SPL* gene expression patterns reveal candidate genes underlying flowering time and architectural differences in Mimulus and *Arabidopsis*. *Mol Phylogenet Evol* **73**, 129-139 (2014).
75. Prunet, N. *et al*. *REBELOTE*, *SQUINT*, and *ULTRAPETALA1* function redundantly in the temporal regulation of floral meristem termination in *Arabidopsis thaliana*. *Plant Cell* **20**, 901-919 (2008).

76. Li, D. *et al*. A repressor complex governs the integration of flowering signals in *Arabidopsis*. *Dev Cell* **15**, 110-120 (2008).
77. Yamaguchi, N. *et al*. A molecular framework for auxin-mediated initiation of flower primordia. *Dev Cell* **24**, 271 – 282 (2013).
78. Jung, J. H., Lee, S., Yun, J., Lee, M. & Park, C. M. The miR172 target *TOE3* represses *AGAMOUS* expression during *Arabidopsis* floral patterning. *Plant Sci* **215-216**, 29-38 (2014).
79. Wahl, V. *et al*. Regulation of flowering by trehalose-6-phosphate signaling in *Arabidopsis thaliana*. *Science* **339**, 704-707 (2013).
80. Cao, Y., Dai, Y., Cui, S. & Ma, L. Histone H2B Monoubiquitination in the Chromatin of *FLOWERING LOCUS C* Regulates Flowering Time in *Arabidopsis*. *Plant Cell* **2586-2602**(2009).
81. Wang, B. *et al*. *UGT87A2*, an *Arabidopsis* glycosyltransferase, regulates flowering time via *FLOWERING LOCUS C*. *New Phytol* **194**, 666-675 (2012).
82. Shi, C., Baldwin, I. T. & Wu, J. *Arabidopsis* plants having defects in nonsense-mediated mRNA decay factors *UPF1*, *UPF2*, and *UPF3* show photoperiod-dependent phenotypes in development and stress responses. *J Integr Plant Biol* **54**, 99-114 (2012).
83. Kerényi, F., Wawer, I., Sikorski, P. J., Kufel, J. & Silhavy, D. Phosphorylation of the N- and C-terminal UPF1 domains plays a critical role in plant nonsense-mediated mRNA decay. *Plant J* **76**, 836-848 (2013).
84. Aluri, S. & Büttner, M. Identification and functional expression of the Arabidopsis thaliana vacuolar glucose transporter 1 and its role in seed germination and flowering. *Proc Natl Acad Sci U S A* **104**, 2537-2542 (2007).
85. Sung, S. & Amasino, R. M. Vernalization in *Arabidopsis thaliana* is mediated by the PHD finger protein VIN3. *Nature* **427**, 159 – 164 (2004).
86. Celesnik, H., Ali, G.S., Robison, F.M. & Reddy, A.S. *Arabidopsis thaliana* VOZ (Vascular plant One-Zinc finger) transcription factors are required for proper regulation of flowering time. *Biol Open* **2**, 424-431 (2013).
87. Gendall, A.R., Levy, Y.Y., Wilson, A. & Dean, C. The *VERNALIZATION 2* gene mediates the epigenetic regulation of vernalization in *Arabidopsis*. *Cell* **107**, 525 – 535 (2001).

Selected references for the gene functions summarized in Supplementary Table S4

1. Krizek, B. A. *AINTEGUMENTA-LIKE* genes have partly overlapping functions with *AINTEGUMENTA* but make distinct contributions to *Arabidopsis thaliana* flower development. *J Exp Bot* **66**, 4537-4549 (2015).
2. Schruff, M. C. *et al*. The *AUXIN RESPONSE FACTOR 2* gene of *Arabidopsis* links auxin signaling, cell division, and the size of seeds and other organs. *Development* **133**, 251–261 (2005).
3. Liu, X. *et al*. *AUXIN RESPONSE FACTOR 3* integrates the functions of *AGAMOUS* and *APETALA2* in floral meristem determinacy. *Plant J* **80**, 629-641 (2014).
4. Choi, K. *et al*. *Arabidopsis* homologs of components of the SWR1 complex regulate flowering and plant development. *Development* **134**, 1931-1941 (2007).
5. Xu, B. *et al*. The *Arabidopsis* genes *AS1*, *AS2* and *JAG* negatively regulate boundary-specifying genes to promote sepal and petal development. *Plant Physiol* **146**, 566-575 (2007).
6. Zhao, D., Yang, M., Solava, J. & Ma, H. The *ASK1* gene regulates development and interacts with the *UFO* gene to control floral organ identity in *Arabidopsis*. *Dev Genet* **25**, 209-223 (1999).
7. Zhao, D., Yu, Q., Chen, M. & Ma, H. The *ASK1* gene regulates B function gene expression in cooperation with *UFO* and *LEAFY* in *Arabidopsis*. *Development* **128**, 2735-2746 (2001).
8. Chae, E., Tan, Q. K., Hill, T. A. & Irish, V. F. An *Arabidopsis* F-box protein acts as a transcriptional co-factor to regulate floral development. *Development* **135**, 1235-1245 (2008).
9. del Pozo, J. C. *et al*. AXR1-ECR1-dependent conjugation of RUB1 to the *Arabidopsis* Cullin AtCUL1 is required for auxin response. *Plant Cell* **14**, 421-433 (2002).
10. del Pozo, J. C. & Estelle, M. The *Arabidopsis* cullin AtCUL1 is modified by the ubiquitin-related protein RUB1. *Proc Natl Acad Sci, USA* **96**, 15342-15347 (1999).
11. Li, G. *et al*. ISWI chromatin remodeling factors and their interacting RINGLET proteins act together in controlling the plant vegetative phase in *Arabidopsis*. *Plant J* **72**, 261-270 (2012).
12. Li, C. L. *et al*. The *Arabidopsis* SWI2/SNF2 chromatin remodeler *BRAHMA* regulates polycomb function during vegetative development and directly activates the flowering repressor gene *SVP*. *Plos Genet* **11**, e1004944 (2015).
13. Aida, M., Ishida, T., Fukaki, H., Fujisawa, H. & Tasaka, M. Genes involved in organ separation in *Arabidopsis*: An analysis of the cup-shaped cotyledon mutant. *Plant Cell* **9**, 841-857 (1997).
14. Calonje, M., Sanchez, R., Chen, L. & Sung, Z. R. *EMBRYONIC FLOWER1* participates in polycomb group-mediated *AG* gene silencing in *Arabidopsis*. *Plant Cell* **20**, 277-291 (2008).
15. Zheng, B. & Chen, X. Dynamics of histone H3 lysine 27 trimethylation in plant development. *Curr Opin Plant Biol* **14**, 123-129 (2011).
16. Chen, Q. *et al*. The *Arabidopsis FILAMENTOUS FLOWER* gene is required for flower formation. *Development* **126**, 2715-2726 (1999).
17. Ratcliffe, O. J., Riechmann, J. L. & Zhang, J. Z. Interfascicular *FIBERLESS1L* is the same gene as Revoluta. *Plant Cell* **12**, 315-317 (2000).
18. Lu, F., Cui, X., Zhang, S., Jenuwein, T. & Cao, X. *Arabidopsis REF6* is a histone H3 lysine 27 demethylase. *Nat Genet* **43**, 715-719 (2011).
19. Magnani, E. & Hake, S. *KNOX* lost the OX: the *Arabidopsis KNATM* gene defines a novel class of *KNOX* transcriptional regulators missing the homeodomain. *Plant Cell* **20**, 875-887 (2008).
20. Hamant, O. & Pautot, V. Plant development: a TALE story. *C R Biol* **333**, 371-381 (2010).
21. Xu, R. & Li, Y. The Mediator complex subunit 8 regulates organ size in *Arabidopsis thaliana*.

Plant Signal Behav **7**, 182-183 (2012).

22. Calonje, M., Sanchez, R., Chen, L. & Sung, Z. R. *EMBRYONIC FLOWER1* participates in polycomb group-mediated *AG* gene silencing in *Arabidopsis*. *Plant Cell* **20**, 277-291 (2008).
23. Running, M. P. *et al*. Enlarged meristems and delayed growth in *plp* mutants result from lack of CaaX prenyltransferases. *Proc Natl Acad Sci U S A* **101**, 7815-7820 (2004).
24. Bao, X., Franks, R. G., Levin, J. Z. & Liu, Z. Repression of *AGAMOUS* by *BELLRINGER* in floral and inflorescence meristems. *Plant Cell* **16**, 1478-1489 (2004).
25. Griffith, M. E., da Silva Conceição, A. & Smyth, D. R. *PETAL LOSS* gene regulates initiation and orientation of second whorl organs in the *Arabidopsis* flower. *Development* **126**, 5635-5644 (1999).
26. Takeda, S., Matsumoto, N. & Okada, K. *RABBIT EARS*, encoding a SUPERMAN-like zinc finger protein, regulates petal development in *Arabidopsis thaliana*. *Development* **131**, 425-434 (2003).
27. Pelaz, S., Ditta, G. S., Baumann, E., Wisman, E. & Yanofsky, M. F. B and C organ identity functions require *SEPALLATA* MADS-box genes. *Nature* **405**, 200-203 (2000).
28. Takahashi, T., Matsuhara, S., Abe, M. & Komeda, Y. Disruption of a DNA topoisomerase I gene affects morphogenesis in *Arabidopsis*. *Plant Cell* **14**, 2085-2093 (2002).
29. Krogan, N. T., Hogan, K. & Long, J. A. *APETALA2* negatively regulates multiple floral organ identity genes in *Arabidopsis* by recruiting the co-repressor TOPLESS and the histone deacetylase HDA19. *Development* **139**, 4180 – 4190 (2012).
30. Roe, J. L., Rivin, C. J., Sessions, R. A., Feldmann, K. A. & Zambryski, P. C. The *Tousled* gene in *A. thaliana* encodes a protein kinase homolog that is required for leaf and flower development. *Cell* **75**, 939-950 (1993).
31. Running, M. P., Fletcher, J. C. & Meyerowitz, E. M. The *WIGGUM* gene is required for proper regulation of floral meristem size in *Arabidopsis*. *Development* **125**, 2545-2553 (1998).
